# Spatial subsetting enables integrative modeling of oral squamous cell carcinoma multiplex imaging data

**DOI:** 10.1016/j.isci.2023.108486

**Published:** 2023-11-20

**Authors:** Jakob Einhaus, Dyani K. Gaudilliere, Julien Hedou, Dorien Feyaerts, Michael G. Ozawa, Masaki Sato, Edward A. Ganio, Amy S. Tsai, Ina A. Stelzer, Karl C. Bruckman, Jonas N. Amar, Maximilian Sabayev, Thomas A. Bonham, Joshua Gillard, Maïgane Diop, Amelie Cambriel, Zala N. Mihalic, Tulio Valdez, Stanley Y. Liu, Leticia Feirrera, David K. Lam, John B. Sunwoo, Christian M. Schürch, Brice Gaudilliere, Xiaoyuan Han

**Affiliations:** 1 Department of Anesthesiology, Perioperative & Pain Medicine, Stanford University School of Medicine, Stanford, CA, USA; 2 Department of Pathology and Neuropathology, University Hospital and Comprehensive Cancer Center Tübingen, Tübingen, Germany; 3 Division of Plastic and Reconstructive Surgery, Department of Surgery, Stanford University School of Medicine, Stanford, CA, USA; 4 Department of Pathology, Stanford University School of Medicine, Stanford, CA, USA; 5 Department of Surgery, Stanford University School of Medicine, Stanford, CA, USA; 6 Division of Pediatrics, Department of Otolaryngology, Stanford University School of Medicine, Stanford, CA, USA; 7 Division of Sleep Surgery, Department of Otolaryngology, Stanford University School of Medicine, Stanford, CA, USA; 8 Department of Oral and Maxillofacial Surgery, University of the Pacific, Arthur A. Dugoni School of Dentistry, San Francisco, CA, USA; 9 Division of Head and Neck Surgery, Department of Otolaryngology, Stanford University School of Medicine, Stanford, CA, USA; 10 Cluster of Excellence iFIT (EXC 2180) “Image-Guided and Functionally Instructed Tumor Therapies”, University of Tübingen, Tübingen, Germany; 11 Department of Biomedical Sciences, University of the Pacific, Arthur A. Dugoni School of Dentistry, San Francisco, CA, USA

**Keywords:** Immunology, Cell biology, Cancer, Machine learning

## Abstract

Oral squamous cell carcinoma (OSCC), a prevalent and aggressive neoplasm, poses a significant challenge due to poor prognosis and limited prognostic biomarkers. Leveraging highly multiplexed imaging mass cytometry, we investigated the tumor immune microenvironment (TIME) in OSCC biopsies, characterizing immune cell distribution and signaling activity at the tumor-invasive front. Our spatial subsetting approach standardized cellular populations by tissue zone, improving feature reproducibility and revealing TIME patterns accompanying loss-of-differentiation. Employing a machine-learning pipeline combining reliable feature selection with multivariable modeling, we achieved accurate histological grade classification (AUC = 0.88). Three model features correlated with clinical outcomes in an independent cohort: granulocyte MAPKAPK2 signaling at the tumor front, stromal CD4^+^ memory T cell size, and the distance of fibroblasts from the tumor border. This study establishes a robust modeling framework for distilling complex imaging data, uncovering sentinel characteristics of the OSCC TIME to facilitate prognostic biomarkers discovery for recurrence risk stratification and immunomodulatory therapy development.

## Highlights

• We developed a robust framework for integrative, multivariable analysis of IMC data
• Subsetting of IMC images into spatial tissue zones improves feature reproducibility
• Cell type-specific immune activation differs between tissue zones in OSCC
• Immune features of OSCC tissue zones are associated with tumor grade and outcomes

## Graphical abstract

**Figure:**
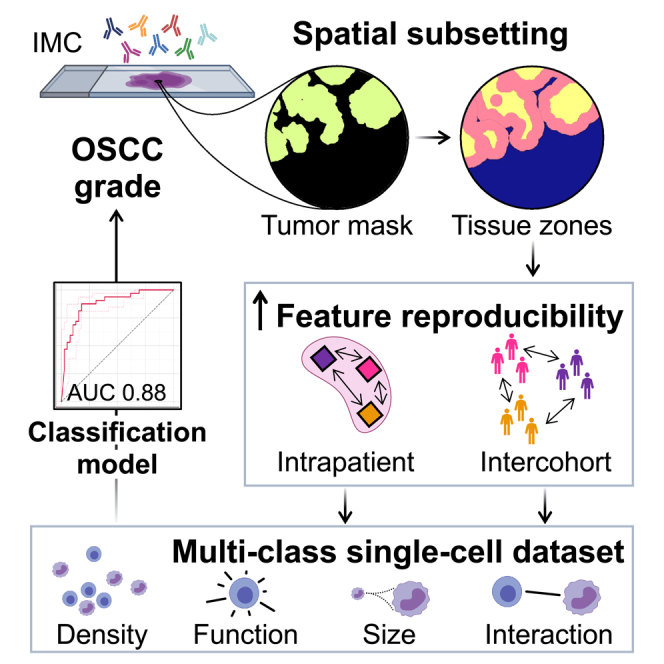

## Introduction

Oral squamous cell carcinoma (OSCC), most frequently presenting on the tongue,^1^ has an aggressive nature (30% recurrence rate) and poor prognosis (50% five-year mortality).^2^ Despite its prevalence, individual prediction of outcomes and prevention of OSCC progression remain challenging.^3^^,^^4^ Prognostic biomarkers are critically needed to determine patients at risk for poor outcomes and can reveal high-yield biological mechanisms as targets for immune modifying therapies. Histopathological grading, which describes the loss of differentiation (i.e., dedifferentiation) of cancer cells based on visual appearance, is an essential component of cancer diagnosis and staging that informs treatment planning and response monitoring. Although grade classification alone is only weakly associated with long-term outcomes, dedifferentiation of cancer cells is an important biological process driving nodal metastasis, tumor recurrence, and ultimately long-term prognosis.^3^^,^^5^^,^^6^^,^^7^ However, the transformative mechanisms of the tumor’s spatial context that elicit tumor cell dedifferentiation is poorly understood.

As OSCC characteristically presents with marked inflammation and heavy immune cell infiltration in the surrounding tissue, crosstalk between tumor cells and the tumor immune microenvironment (TIME) plays an important role in dedifferentiation and cancer progression.^8^^,^^9^^,^^10^ Innate immune cells, such as M1 macrophages, tumor associated granulocytes, and classical monocytes, are known to exert anti-tumor effects with increased proinflammatory cytokines, including IL-1β, IL-6, and TNF-α, and reactive oxygen species.^11^^,^^12^ However, these immune defense mechanisms can also induce tumor cell dedifferentiation and OSCC invasion through activation of anti-apoptotic and cell cycle-regulating genes in tumor cells.^13^^,^^14^ Sequentially, tumor dedifferentiation can reduce adaptive immune cells’ tumor surveillance functionality, e.g., by promoting T cell exhaustion and immunosuppressive activity of regulatory T cells via IL-4, IL-10, and TFG-β.^15^^,^^16^ This complex interplay of tumor-immune interactions requires careful contextual interpretation. Previous studies have highlighted individual immune drivers of dedifferentiation and identified TIME correlates of outcomes, such as treatment response^17^ or disease-free survival and mortality.^18^^,^^19^^,^^20^ However, limitations in the number of cellular features that can be assessed simultaneously have thus far precluded an integrative analysis of phenotypic, functional, or spatial features. As a result, important single-cell TIME characteristics linked to clinically relevant endpoints remained undetected.

Highly multiplexed imaging approaches, such as imaging mass cytometry (IMC), have transformed our ability to study complex and heterogeneous tissues by allowing simultaneous measurement of over 50 histological markers per single cell.^21^^,^^22^^,^^23^ Paired with advances in computational image processing, these technologies enable comprehensive phenotypic and functional characterization of multiple cell subsets with unprecedented spatial resolution and generate high-dimensional datasets containing thousands of single-cell features.^24^^,^^25^ However, rigorous statistical approaches that integrate the resulting large number of phenotypic, functional, and spatial features into reliable multivariable models are critically lacking. As such, investigators often rely on univariate analyses, which are ill-adapted given the high number of measured features relative to the number of biospecimens analyzed.^26^^,^^27^

Here, a highly multiplexed IMC approach was employed to comprehensively characterize the phenotypic and functional landscape of the TIME in tissue biopsies from patients with OSCC. We hypothesized that IMC features stratifying OSCC grade would unveil distinct TIME characteristics associated with tumor dedifferentiation that would have been undetected using traditional imaging techniques. The first aim of the study was to create a spatial, phenotypic, and functional characterization of OSCC tissue architecture in distinct tissue compartments. To this end, we developed a robust analysis framework that implemented spatial subsetting of the tissue into stroma, tumor front, and tumor core, and tested the reliability and reproducibility of these spatial classifiers. The second aim was to identify statistically reliable TIME correlates of OSCC grade using a robust multivariable framework with validation of model features in an independent cohort. Finally, we tested whether a subset of model features correlated with long-term clinical outcomes, available for one of the two cohorts.

## Results

### Mapping the tumor immune landscape in OSCC using imaging IMC

**Figure 1:**
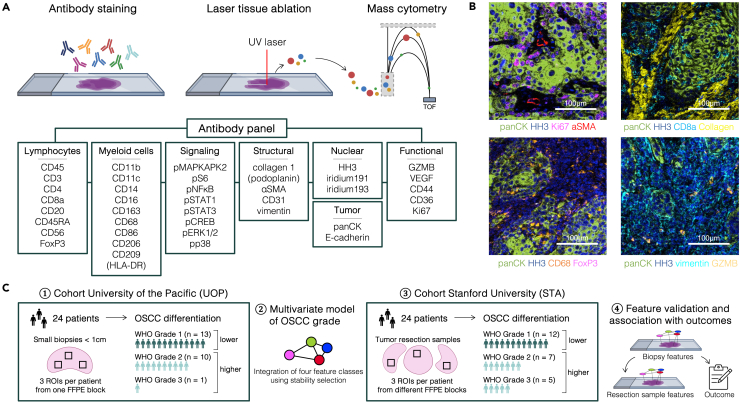
Analysis of the tumor immune microenvironment (TIME) in oral tongue squamous cell carcinoma (OSCC) using imaging mass cytometry (IMC) (A) Experimental workflow: Tumor samples of invasive OSCC were stained with a 40-plex IMC antibody panel, then laser-ablated and analyzed using a Hyperion imaging system. (B) Representative staining examples of regions of interest (ROIs) at the tumor (panCK^+^) invasive front. (C) Analysis workflow: IMC data were generated from OSCC incisional biopsies from 24 patients at University of the Pacific (UOP cohort) and used to build a multivariable model of OSCC grade. In an independent cohort of OSCC resection specimens from 24 patients at Stanford (STA cohort), model features identified in the UOP cohort were validated for their association with tumor grade and analyzed post-hoc for association with clinical outcomes. GZMB = granzyme B, HH3 = histone H3, panCK = pancytokeratin.

A 40-plex antibody panel was developed and optimized to characterize the phenotypic, functional, and spatial organization of the TIME in OSCC (Table S1). The panel, containing three nuclear, five structural, 14 functional, and 18 phenotypic markers, allowed for detection of major innate and adaptive immune cell populations, fibroblasts, endothelial cells, and tumor cells, as well as intracellular activities (i.e., phosphorylation state) of eight signaling proteins (Figure 1A). Regions of interest (ROIs) were selected along the tumor invasive front to explore distinct interaction patterns between the tumor cells (panCK^+^) and their stromal and immune cell surroundings (Figure 1B). This study included two independent patient cohorts from two institutions. The University of the Pacific Arthur A. Dugoni School of Dentistry (UOP) cohort included formalin-fixed, paraffin-embedded (FFPE) incisional oral tongue biopsies from 24 patients with OSCC for IMC analysis. Samples were selected in chronological order by date of resection and included 13 well differentiated (WHO grade 1) samples, 10 moderately differentiated (WHO grade 2) samples, and one poorly differentiated (WHO grade 3) sample based on the grading in the original pathology reports. Due to the low number of grade 3 samples in the cohort, consistent with the lower incidence of grade 3 tumors as reported in the literature,^28^ the biopsies were grouped into one of two classes for analysis: lower grade (WHO grade 1) or higher grade samples (WHO grade 2 or 3, Figure 1C). Using IMC data from 71 ROIs, a multivariable model of OSCC tumor grade was constructed. The second cohort of 24 tumor specimens (12 well, 7 moderately, and 5 poorly differentiated) from primary, treatment-naïve OSCC resection cases was collected at Stanford University Hospital (STA). Basic demographic information was recorded for both cohorts, and patient outcomes data, including three-year recurrence and five-year mortality, as well as adjuvant treatment modalities were available and recorded for the STA cohort only (Tables S2 and S3). By design, ROIs in the biopsy samples from the UOP cohort were confined to smaller biospecimens than ROIs in the resection samples of the STA cohort which allowed for sampling of disparate regions of the tumor.

### Immune cell populations at the tumor invasive front of OSCC

**Figure 2:**
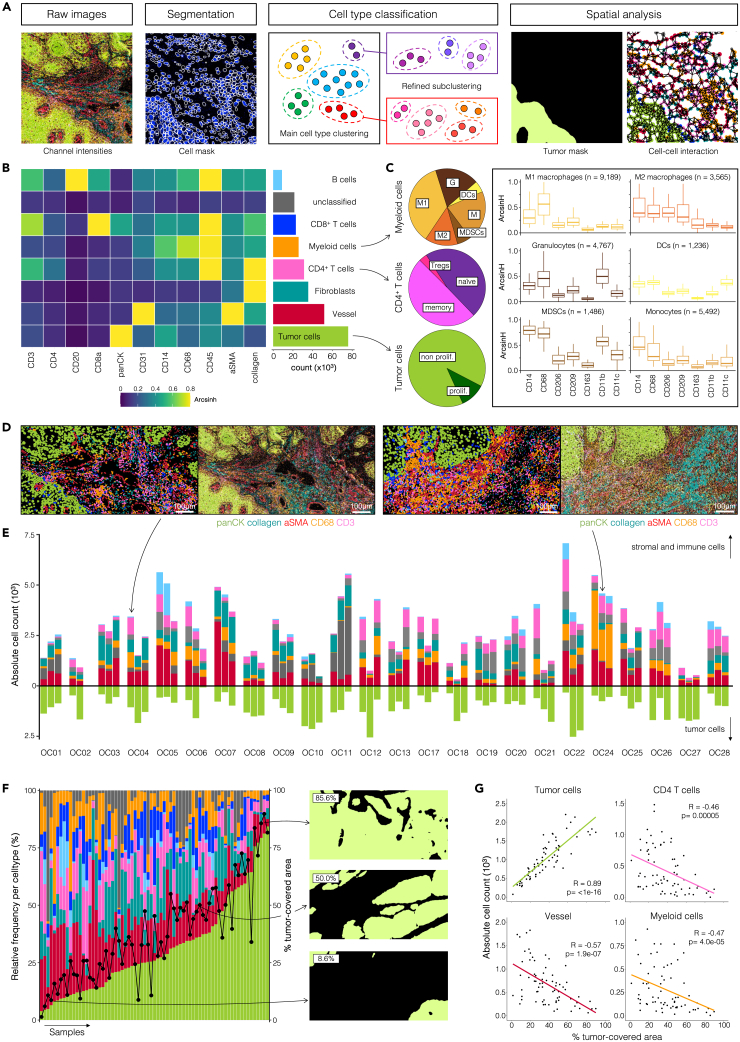
Cell composition of the TIME in OSCC (A) Analytical pipeline for single-cell and spatial feature extraction from IMC data. Cell segmentation was performed using Mesmer and cell populations were identified using iterative PhenoGraph clustering. Spatial analysis was based on the generation of tumor masks and the extraction of cell-cell interaction data. (B) Heatmap of mean marker expression levels and absolute count of major cell subsets. (C) Relative distribution of myeloid (*upper left*), CD4^+^ T cell (*middle left*), and tumor cell (*lower left*) subpopulations. Mean marker expression levels of the respective myeloid subpopulations (*right*). Boxes indicate median and interquartile range (IQR), whiskers indicate 1.5xIQR. (D) Pseudo-coloring of two representative ROIs, color-coded as in B (*left panels*), in comparison to the raw image of panCK, collagen, aSMA, CD68, and CD3 expression (*right panels*). (E) Intra- and interpatient variability in cell abundance across ROIs. Depicted are absolute cell counts per major cell subset. (F) Relative frequency (proportion of total cell count) per major cell subset for all samples ordered by increasing tumor cell frequency; black dots represent the proportion of tumor-covered area per ROI (*left panel*). Examples of tumor masks varying in tumor-covered area (*right panels*). (G) Spearman correlation between absolute cell count and percentage of tumor-covered area.

In each cohort, 71 ROIs were analyzed using a Hyperion imaging system. Single-cell segmentation of the resulting 40-plex images was performed using the deep-learning algorithm Mesmer^29^ on two channel images of selected membrane and nuclear markers (Figure 2A; Table S1). The produced single-cell datasets comprised 273,408 (UOP) and 224,830 (STA) cells after excluding segmented objects smaller than 10 pixels and with a DNA1/DNA2 signal under or over two standard deviations from the mean. Coarse cell phenotyping was performed using PhenoGraph^30^ on 12 major lineage markers (aSMA, CD3, CD4, CD8, CD14, CD20, CD31, CD45, CD68, collagen, pancytokeratin, and vimentin). The identified PhenoGraph clusters were assigned to seven major populations (Figures 2B, S1): tumor cells (panCK^+^), CD4^+^ T cells (CD45^+^CD3^+^CD4^+^), CD8^+^ T cells (CD45^+^CD3^+^CD8^+^), B cells (CD45^+^CD20^+^), blood vessel (CD31^+^ and/or aSMA^+^), fibroblasts (collagen^+^), and myeloid cells (CD45^+^CD14^+^). Cells that showed ambiguous marker expression patterns were labeled as unclassified. More detailed phenotyping of subpopulations was subsequently performed by iterating PhenoGraph clustering on three of the aforementioned major subsets, namely CD4^+^ T cells (regulatory = FoxP3^+^, naive = CD45RA^+^, memory = CD45RA^−^), tumor cells (proliferating = Ki67^+^, non-proliferating = Ki67^-^), and myeloid cells (M1 macrophages = CD68^+^CD206^-^, M2 macrophages = CD68^+^CD163^+^CD206^+^, monocytes = CD14^+^CD68^low^, granulocytes = CD14^low^CD11b^+^Granzyme B^+^, dendritic cells = CD11c^+^, myeloid derived suppressor cells [MDSCs] = CD14^+^CD11b^+^HLA-DR^-^, Figure 2C, see STAR Methods). To confirm the identity of the 15 cell types defined through unsupervised clustering, raw signal images and annotated cell masks across patients were compared visually and showed high concordance (Figures 2D, S2, and S3).

Examination of the phenotypic organization across the UOP OSCC biopsies revealed that of the 15 cell subsets characterized, the most abundant cell population was non-proliferating tumor cells (24.8%), while immune infiltrates were mainly composed of CD8^+^ T cells (8.4%), memory CD4^+^ T cells (5.7%), and M1 macrophages (3.4%). M2 macrophages (1.3%), MDSCs (0.5%), and regulatory T cells (0.4%) were the least abundant cell types. An additional 7.8% of cells remained unclassified (Figure 2B). The STA OSCC resection samples showed a similar distribution of cell populations, with higher proportions of innate immune cells and fewer structural cells (vessel, fibroblasts) and unclassified cells (2.1%, Figure S1). The differences in the distribution and expression pattern of the unclassified cells between the two cohorts point to different causes of non-classification: fixation artifacts, low expression markers, or cell segmentation impurities. However, the presence of unclassified cells did not hinder further multivariable analyses and predictive model accuracy.

Quantification of the absolute cell number for individual subpopulations provided a global overview of immune and tumor cell distribution at the OSCC tumor front. High variability in the cellular composition of ROIs was observed between patients (interpatient variability) and between ROIs from each patient (intrapatient variability) in both cohorts (Figures 2E and S1). Notably, the proportion of tumor-covered area differed substantially between ROIs, thereby contributing to variability between samples without representing informative biology (Figure 2F). As such, the abundance of major cell types (e.g., tumor, vessel, myeloid, and CD4^+^ T cells) strongly correlated with the proportion of tumor-covered area per ROI (Figure 2G). Variability between ROIs can result from either a sampling error (random variability) or from underlying tissue heterogeneity (biological variability). Reducing uninformative variability in IMC data represents an important challenge in the analysis of multiparametric imaging studies. Therefore, further analysis steps were taken to ensure sample comparability for meaningful interpretation of biological differences—whether phenotypically, functionally, or spatially—on a cell population level.

### Spatial subsetting of OSCC images into stromal, tumor front, and tumor core zones enables meaningful comparisons between heterogeneous samples

**Figure 3:**
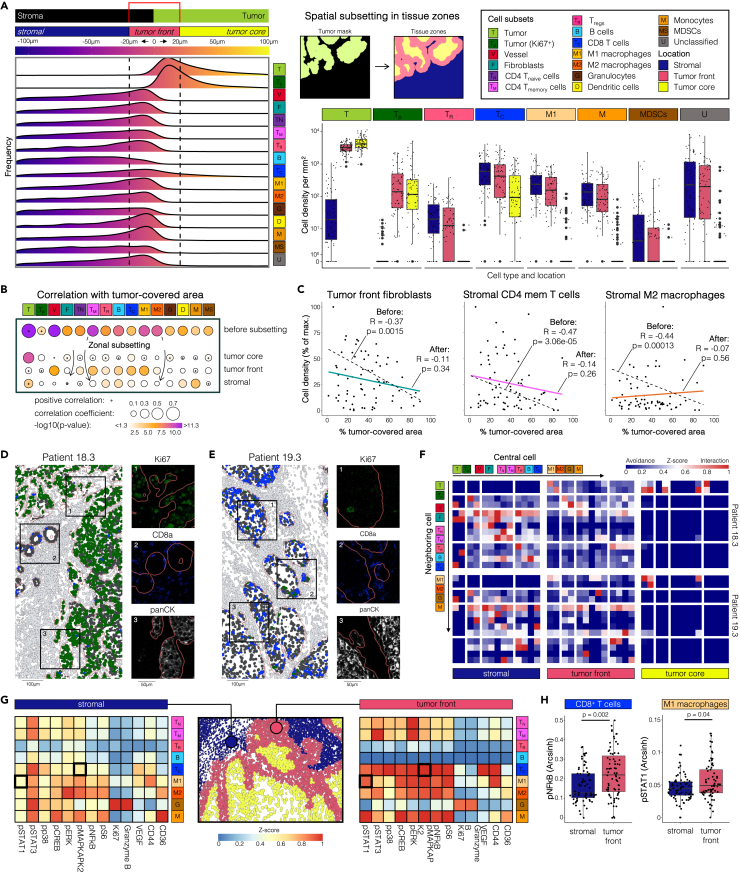
Spatial subsetting of OSCC images reveals spatial characteristics of the stroma, tumor front, and tumor core (A) Spatial distribution of cell populations relative to the tumor border (*left*). Zonal densities of cell populations in all ROIs in the stroma, at the tumor front, and in the tumor core (*right*). (B) Spearman correlation between the cell population density of each cell type and the proportion of tumor-covered area before and after spatial subsetting. Correlation coefficients are negative unless indicated otherwise. (C) Examples of reduction in cell density bias (i.e., correlation between cell density and tumor-covered area) achieved by spatial subsetting as indicated with arrows in B. (D and E) Pseudocoloring of CD8^+^ T cells and proliferating tumor cells of two contrasting samples, color-coded as in panel B and depicting the tumor border (red line). Only cells within the tumor border are color-coded, while cells external to the tumor are grayed out. Image subsections show the original staining for markers of tumor cells (panCK), CD8^+^ T cells (CD8a), and proliferating tumor cells (Ki67) at higher resolution. Scale bars indicate 100 μm for the entire ROI and 50 μm for magnified subsections, respectively. (F) Mean neighborhood coefficients of zonal cell-cell interactions per cell type reflect infiltration patterns for patients in (D) and (E). The coefficient indicates the proportion of neighboring cells from each cell type for a given center cell. (G) Heatmap of z-scored mean functional marker expression levels in all samples across immune cells in the stroma and at the tumor front. (H) Differences in signaling activity of pNFκB in CD8^+^ T cells and pSTAT1 in M1 macrophages between stromal and tumor front zones as highlighted in (G). Boxes indicate median and interquartile range (IQR), whiskers indicate 1.5xIQR. Significance was calculated using a two-sided Mann-Whitney test.

To investigate inherent differences in cell population distribution and localization patterns within ROIs and correct for the confounding effect of variability in tumor-covered area on cell population densities (Figure 3A), a random forest pixel classifier was trained to generate tumor masks and derive tumor and stromal area in mm^2^ (Figure 3A). Boolean conversion of the resulting binary image provided a Euclidean distance value from the tumor border to each cell’s border, allowing for characterization of the spatial distribution of cell types relative to the tumor border.^31^ Each cell was then assigned to one of three zones, representing three major and biologically diverse environments: tumor core (greater than 20 μm into the tumor), tumor front (within 20 μm of the tumor border), and stroma (greater than 20 μm away from the tumor). The definition of tumor front cells within 20 μm of distance was consistent with the employed definition of neighboring cells of five nearest neighbors within 20 μm of Euclidean distance. Interestingly, this definition also included maximum cell frequencies relative to the tumor border, indicating the cell-rich nature of this biological compartment. The zonal density of each cell population was calculated for each zone by dividing the absolute number of cells within the population by the respective area of each zone (stroma, tumor front, or tumor core, Figures S4 and S5). This spatial subsetting approach enabled meaningful comparisons of cell population densities per zone within and across samples (Figure 3B). Notably, CD8^+^ T cells were the only immune cell population with consistent tumor infiltration (Figure 3A), while other immune cells, such as CD4^+^ memory T cells, granulocytes, or M1 macrophages, were found within the tumor boundaries in only 15.5%, 8.4%, and 23.9% of the samples respectively (Figures S4 and S5). Proliferating tumor cells were more densely distributed at the tumor front compared to the tumor core, whereas MDSCs were predominantly found in the stroma.

To assess the validity of this approach, we compared the correlation of cell population densities with tumor-covered area per ROI before and after spatial subsetting. Before subsetting, the abundance of all 15 UOP and 13 of 15 STA cell populations significantly correlated with tumor-covered area (Figures 3B and S1), which was effectively mitigated after spatial subsetting (Figure 3C). Thereby, zonal cell densities accurately reflected contrasting cell distribution patterns within distinct tissue environments, highlighting biologically meaningful differences between ROIs, for example in the density of tumor-infiltrating CD8^+^ T cells and proliferating tumor cells (Figures 3D and 3E).

Furthermore, spatial subsetting revealed important regional differences in cell-cell interaction (Figure 3F) and cell function (Figure 3G) features. As depicted on the zonal neighborhood heatmaps (Figure 3F), each zone displayed a unique fingerprint of neighborhood relationships between cell populations. From a functional standpoint, striking differences were observed in immune cell signaling activities between the tumor front and stroma, which were cell-type specific, including in M1 and M2 macrophages as well as in CD8^+^ T cells (Figure 3G). Specifically, activation of CD8^+^ T cell MyD88 and JAK/STAT signaling was higher in closer proximity to the tumor, and M1 macrophages and monocytes showed stronger activation of pSTAT1 at the tumor front (Figure 3H). Conversely, compared to tumor front granulocytes, stromal granulocytes expressed higher levels of granzyme B and Ki67. These examples highlight how the subsetting approach exposed contrasting functional features between biologically distinct environments that would have otherwise remained undetected.

In summary, spatial subsetting improved comparability between OSCC tissue samples by reducing feature bias resulting from the variability of tumor-covered area per ROI. Ultimately, this approach fine-tuned the spatial analysis of the IMC dataset, providing a granular view of individual cell phenotypes and functional activation states relative to their zonal location in the stroma, tumor front, or tumor core.

### Spatial subsetting increases inter-cohort and intrapatient feature reproducibility when analyzing tumor samples of varying size

**Figure 4:**
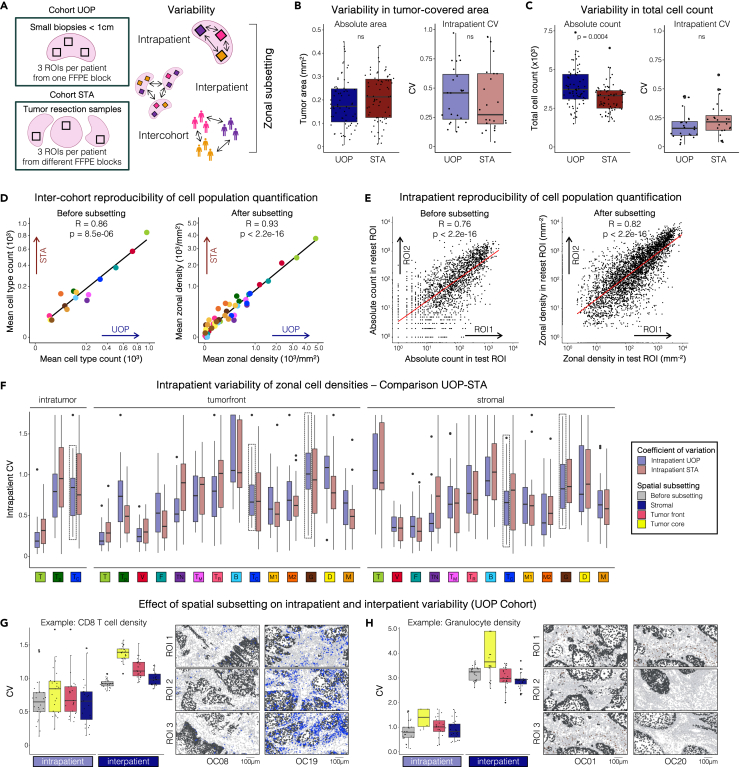
Spatial subsetting increases intrapatient and inter-cohort feature reproducibility in OSCC biopsies and resection samples (A) Levels of heterogeneity (i.e., intrapatient, interpatient, and inter-cohort variability) assessed in this study. ROIs originated from incisional OSCC biopsies (UOP cohort) or resection specimens (STA cohort). (B) Differences between cohorts in the confounding variable of tumor-covered area per ROI. Absolute tumor-covered area (*left*) and its intrapatient coefficient of variation (CV) as an estimation of variability between ROIs from one individual patient (*right*) are depicted. (C) Differences between cohorts in the confounding variable of total cell count per ROI. Absolute cell count (*left*) and its intrapatient coefficient of variation (CV) as an estimation of variability between ROIs from one individual patient (*right*) are depicted. (D) Inter-cohort reproducibility of cell population quantification before and after spatial subsetting. Spearman correlation is shown for the comparison of mean absolute cell type counts of all 15 cell populations between the two cohorts (*left*) and for the comparison of mean zonal cell population density of all 15 cell populations across the three tissue zones between the two cohorts (*right*). (E) Intrapatient reproducibility of cell population quantification before and after spatial subsetting. Spearman correlation is shown for dataset-wide pairwise comparisons of absolute cell population count of all cell populations between ROIs from the same patient (*left*) and for dataset-wide pairwise comparisons of zonal cell population densities between ROIs of each patient (*right*). (F) Intrapatient CVs of all cell population densities comparing the UOP and STA cohort. (G) Comparison of intra- and interpatient CVs for CD8^+^ T cells before and after spatial subsetting (*left*). Cell masks colored for CD8^+^ T cells of representative ROIs from two patients (*right panels*). (H) Comparison of intra- and interpatient CV for granulocytes before and after spatial subsetting (*left*). Cell masks colored for granulocytes of representative ROIs from two patients (*right panels*). Scale bars indicate 100 μm. Boxes indicate median and interquartile range (IQR), whiskers indicate 1.5xIQR. Significance was calculated using a two-sided Mann-Whitney test.

In multiplexed tissue imaging, selecting multiple small ROIs within a tissue sample is a common approach to overcome constraints in data size and complexity, acquisition time, or tissue availability. However, this strategy poses considerable challenges in comprehensively capturing tissue heterogeneity and ensuring the relevance of selected ROIs for patient-level observations. The biospecimens in our study included both OSCC incisional biopsies (UOP) and tumor resection samples (STA), providing a unique opportunity to quantify important reproducibility and variability metrics applicable to other IMC studies. By comparing IMC features between cohorts, between patients, and between ROIs from each individual patient (Figure 4A), we evaluated the effect of spatial subsetting on the reproducibility of IMC features and quantified intra- and interpatient variability.

In both cohorts, ROI selection followed the criteria of including regions along the tumor invasive front to capture tumor tissue and the adjacent stroma. Two unsystematic, outcome-independent variables related to ROI selection confounded cell subset density values: the proportion in tumor-covered area and, to a lesser extent, the total cell count per ROI. In both cohorts, the proportion of tumor-covered area displayed a wide range across ROIs. Importantly, the mean proportion across all ROIs from each cohort (37.5% and 42.2% for UOP and STA cohorts, respectively) and its intrapatient variability remained consistent for both cohorts (Figure 4B). Regarding total cell count per ROI, UOP biopsy samples exhibited higher counts on average (3,950 cells) compared to STA resection samples (3,166 cells), while intrapatient variability in total cell count was consistent between the two cohorts (Figure 4C). In summary, the variables confounding cell subset densities did not show higher heterogeneity when ROIs were selected from disparate regions of larger tissue samples.

Correlation analyses were performed to determine whether spatial subsetting improved the reproducibility of cell population measurements between cohorts and between ROIs for each individual patient. Comparisons between the STA and UOP cohorts showed that the correlation in mean zonal densities for each cell population after spatial subsetting (R = 0.93, Figure 4D) was higher than the correlation in mean cell type abundance obtained before spatial subsetting (R = 0.86), indicating higher inter-cohort reproducibility after subsetting. Similarly, pairwise comparison of cell population quantifications between two ROIs from the same patient showed that spatial subsetting also increased intrapatient reproducibility (Figure 4E, R = 0.82 for zonal cell population densities after subsetting vs. R = 0.76 for cell population abundance before subsetting). This effect was even more pronounced when focusing on each biological cell subset individually (Figure S6). In conclusion, spatial subsetting effectively improved inter-cohort and intrapatient reliability and reproducibility of cell population quantification.

To support predictive modeling and patient-level assumptions based on individual images, IMC studies depend on interpatient variability surpassing intrapatient variability. Thus, we compared the intra- and interpatient variability of zonal cell densities between the UOP and STA cohorts. Between the two cohorts, intrapatient variability of zonal cell densities did not differ, indicating that OSCC biopsies and resection samples indeed showed similar levels of heterogeneity in this regard (Figure 4F). Between patients, variability in cell population density differed between the two cohorts for only a few zonal cell densities (e.g., monocytes and granulocytes in the stroma, Figure S7). Importantly, for all cell populations, interpatient variability remained higher than intrapatient variability. Spatial subsetting resulted in only minimal changes to the relationship between inter- and intrapatient variability, increasing inter-relative to intrapatient variability for certain cell populations (e.g., tumor core CD8^+^ T cells, Figure 4G) and reducing it for others (e.g., stromal granulocytes, Figure 4H). Ultimately, intrapatient variability levels did not differ between IMC cohorts using smaller (UOP) or larger (STA) tissue samples and spatial subsetting preserved the intrapatient and interpatient heterogeneity structure of IMC data.

### Integrative multivariable modeling of TIME features accurately classifies OSCC tumor grade

**Figure 5:**
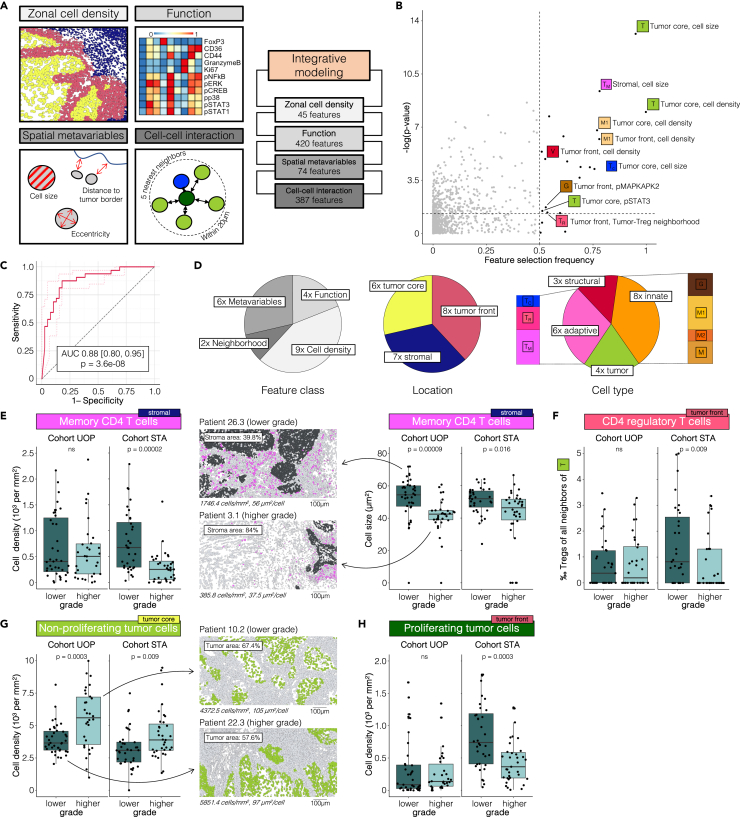
Integrative multivariable modeling of TIME features accurately classifies OSCC tumor grade (A) Integration of four feature classes (zonal cell density, function, neighborhood, and spatial metavariables) using stability selection. Individual LASSO models were built on each feature class (i.e., 45 zonal density values, 420 functional features, 74 spatial metavariables, and 387 neighborhood coefficients per sample) separately before integrating selected model features in a final logistic regression model. (B) Volcano plot depicting feature selection frequency across bootstrapping LASSO iterations and univariate p value (Mann-Whitney U test). (C) Area under the receiver operator curve (AUROC) plot for the final multivariable model. (D) Model feature origin by feature class (*left*), zonal location (*middle*), and cell type (*right*). (E) Differences in cell density (*left*) and cell size (*right*) of stromal CD4^+^ memory T cells between lower and higher grade. Representative patient examples of cell masks pseudocolored for stromal CD4^+^ memory T cells (*middle*). (F) Differences in the neighborhood coefficient of tumor front regulatory CD4^+^ T cells and non-proliferating tumor cells between lower and higher grade. (G) Differences in the cell density of tumor front proliferating tumor cells between lower and higher grade. (H) Differences in cell density of tumor core non-proliferating tumor cells between lower and higher grade. Representative patient examples of cell masks pseudocolored for tumor core non-proliferative tumor cells. Scale bars indicate 100 μm. Boxes indicate median and interquartile range (IQR), whiskers indicate 1.5xIQR. Univariate p values were calculated using a two-sided Mann-Whitney test.

Systematic feature extraction from our multiplexed analysis of OSCC samples provided a high-dimensional dataset containing four distinct feature classes reporting information per spatial tissue zone, including zonal cell densities (45 features), cell type-specific functional activation (420 features), single-cell spatial metavariables (74 features), and cell-cell interaction (374 features, see STAR Methods, Figure 5A).

A sparse machine learning method was implemented to integrate the four feature classes into a multivariable predictive model classifying the UOP OSCC biopsy samples as lower or higher grade, effectively stratifying features according to the tumors’ loss of differentiation. Using a bootstrapping and cross-validation procedure adapted from stability selection, least absolute shrinkage and selection operator (LASSO) models were built on each feature class individually (Figure S8). Features with a selection frequency larger than 0.5 across all bootstrap iterations were chosen from each feature class (Figure 5B). Within each cross-validation fold of the deployed Monte Carlo cross-validation, a logistic regression model was built on the threshold-passing features from all four feature classes, and model performance was evaluated on the samples held out for testing. Within the cross-validation procedure, the integrative model classifying histological grade demonstrated high accuracy, quantified by the area under the receiver-operating characteristic curve (AUC = 0.88, 95% CI [0.8, 0.95], p = 3.6e-08, Figure 5C). The final model, built on the entire dataset for feature selection, included nine cell population densities, six spatial metavariables, four functional features, and two neighborhood coefficients classifying OSCC grade (Figure 5D). Notably, immune cell features comprised 67% of model features. Additional model features included four tumor cell and three structural features. Differences in individual model features between higher and lower grade were tested independently using samples from the STA cohort (Figures S9–S11).

The majority of the features selected by the model were immune cell features characterizing the abundance or function of specific innate and adaptive immune cells. CD4^+^ memory T cells were found to be less abundant and smaller in size in the stroma of higher-grade samples (Figure 5G). The same trend was also observed for CD8^+^ T cells in the tumor core. At the tumor front, increased cell-cell interactions between regulatory CD4^+^ T cells and non-proliferating tumor cells were indicative of well differentiated tumors (Figure 5H). Multiple innate immune cell features showed association with higher grade, such as increased M1 macrophage density, reduced distance of monocytes from the tumor, and increased Ki67 levels in granulocytes in the stroma (Figure S9). At the tumor front, granulocytes showed reduced pMAPKAPK2 signaling activity in higher grade tumors.

An informative tumor cell feature differentiating tumor grade was the density of non-proliferative tumor cells in the tumor core (Figure 5G). The tumor cell density was increased in higher grade tumor samples, which was also consistent with a distinctly smaller tumor cell size in higher grade tumors. Another tumor cell feature selected by the model was a reduced cell density of proliferating tumor cells at the tumor front in higher grade tumors (Figure 5H). The abundance of tumor cells found in the stroma did not differ between histological grades. Finally, several structural features, such as vessel cell density and eccentricity at the tumor front and the distance of fibroblasts from the tumor border, differentiated OSCC tumors by grade.

Of the features selected by the model built on samples from the UOP cohort, five independently validated on samples from the STA cohort including CD4^+^ T cell size and density in the stroma, neighborhood relationship between regulatory CD4^+^ T cells and non-proliferating tumor cells, and cell densities of non-proliferating tumor cells in the tumor core and proliferating tumor cells at the tumor front (Figures 5 and S10).

### A subset of features associated with OSCC tumor grade correlate with long-term clinical outcomes

**Figure 6:**
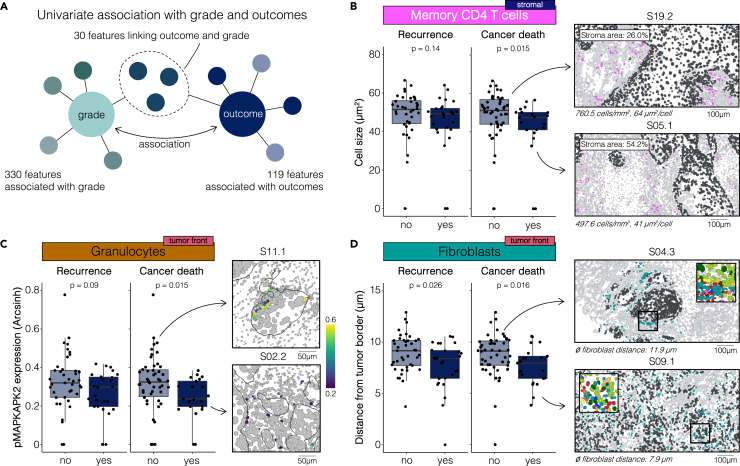
A subset of features associated with OSCC tumor grade correlates with long-term clinical outcomes (A) Scientific hypothesis of the analysis workflow, illustrating quantification of features associated with grade, outcomes, or both (uncorrected two-sided Mann-Whitney test, p < 0.05). (B) Differences in cell size of stromal CD4^+^ memory T cells at the tumor front between patients with and without tumor recurrence or cancer-related mortality (*left*). Representative patient examples of cell masks colored for CD4^+^ memory T cells (*pink*) and tumor (*dark gray*) cells (*right*). Scale bars indicate 100 μm. (C) Differences in mean pMAPKAPK2 expression of granulocytes at the tumor front between patients with and without tumor recurrence or cancer-related mortality (*left*). Representative patient examples of cell masks with granulocytes colored by pMAPKAPK2 expression (*right*). Scale bars indicate 50 μm. (D) Differences in fibroblasts' distance from the tumor border at the tumor front between patients with and without tumor recurrence or cancer-related mortality (*left*). Representative patient examples of cell mask pseudocoloring for tumor front fibroblasts (*right*). Insets (2x) show all cell subsets. Scale bars indicate 100 μm. Boxes indicate median and interquartile range (IQR), whiskers indicate 1.5xIQR. Univariate p values are calculated using a two-sided Mann-Whitney test.

In clinical routine, tumor grade is typically assessed through visual inspection of H&E-stained tumor slides, offering critical insights into the aggressivity and biological characteristics of the tumor. We reasoned that TIME features, such as organization and function of immune and structural cells, that vary with the tumor cells’ loss-of-differentiation could represent biological factors that also contribute to tumor recurrence and cancer-related mortality, thereby linking the pathophysiology of OSCC grade and clinical outcomes (Figure 6A). Hence, we investigated whether the features identified by the model constructed based on OSCC grade were associated with clinical outcomes in the STA cohort. Three of the model features were associated with tumor recurrence within three years and/or cancer-related mortality within five years after diagnosis. Interestingly, all three features were characteristics of immune and structural cells located at the tumor front or in the stroma. Decreased CD4^+^ memory T cell size in the stroma was associated with cancer-related mortality (Figure 6B). At the tumor front, cases with at least five-year cancer free survival displayed increased pro-inflammatory pMAPKAPK2 signaling activity in granulocytes (Figure 6C). Finally, closer contact of fibroblasts with the tumor border was associated with both recurrence and cancer-related mortality (Figure 6D). Visual examination of exemplary cases with worse outcomes revealed a maintained fibroblast wall around the tumor. In contrast, cases with at least five-year survival showed immune cells frequently breaching the fibroblast wall around the tumor.

## Discussion

The need for clinical biomarkers reflecting tumor aggressiveness and disease progression to personalize therapeutic management and improve outcomes for patients with OSCC is critical. Toward this end, our study utilized IMC to provide in-depth and functional characterization of the spatial relationships between tumor, stroma, and immune cells in patients with OSCC. However, analysis of multiplexed imaging data presents considerable challenges, including feature reproducibility and dataset dimensionality. To address these issues, we introduced a spatial subsetting approach to standardize cell population metrics to tissue zones, extracted four reproducible feature classes that integrated single-cell and spatial information to derive a curated feature set amenable to multivariable modeling, and used a robust machine learning pipeline to stratify OSCC tumors by grade.

Variability between ROIs in multiplexed imaging studies can be attributed either to random variability due to the sampling process or to biological variability indicating underlying tissue heterogeneity. Our results show that spatial subsetting, achieved by combining existing tools for high dimensional IMC data analysis,^24^^,^^29^^,^^31^^,^^32^ is an effective approach to reduce bias introduced by variability in tumor-covered area, an inherent byproduct of ROI selection. Most importantly, spatial subsetting improved intrapatient reproducibility of cell population densities between ROIs, enabling relevant patient-level inferences based on individual images. Additionally, spatial subsetting also enhanced the consistency of cell population features between cohorts sampling OSCC tissue samples of varying sizes. Our findings indicate that spatial subsetting is particularly appropriate when conducting predictive modeling analyses to enhance the reproducibility and generalizability of sample-level (and patient-level) observations. Interestingly, the remaining intrapatient heterogeneity in cell population densities after spatial normalization was as high in OSCC biopsies as in resection samples.

The spatial subsetting analysis also shed light on the compartmentalized tissue biology within the highly inflamed surroundings of OSCC tumors.^10^^,^^33^ Anti-tumor immune cells such as CD8^+^ T cells and M1 macrophages showed higher activation in MyD88 and JAK/STAT signaling pathways closer to the tumor front, whereas immunoregulatory cell populations, such as M2 macrophages, regulatory T cells, and dendritic cells showed higher activation in the stroma. These findings are consistent with the concept that pro-inflammatory and immunoregulatory immune activities occur simultaneously in spatially contrasting compartments of the tumor-adjacent stroma^34^: Immune cells in close contact with the tumor may maintain their tumoricidal phenotype and function, while the stromal predominance of immunosuppressive subsets may reduce further recruitment of immune cells.

IMC datasets comprise multiple feature classes (e.g., zonal cell density, function, neighborhood, and spatial metavariables) of varying dimensionality and data structure. As feature numbers typically far exceed the number of samples, traditional univariate and multivariable approaches remain ill-adapted for IMC data analysis.^35^^,^^36^^,^^37^^,^^38^ To account for the dimensionality and multilayered nature of the data, we employed a stringent statistical framework that combined stability selection and late fusion logistic regression modeling within a bootstrapping and cross-validation procedure. The analysis identified a set of phenotypic, functional, and spatial TIME features that accurately classified OSCC histological grade, a subset of which validated in an independent cohort.^39^^,^^40^ The late-fusion nature of this modeling approach produced a classification model well balanced between the feature classes and biological compartments.

Four of the selected model features represented tumor cell characteristics: non-proliferating tumor cells were smaller in size and denser in higher grade tumors. This finding demonstrated the ability of IMC to capture cell size as an important measure of tissue aberration in malignancies.^41^ The reduced tumor cell size may be an indicator of accelerated, compacting tumor growth. Additionally, in higher grade tumors, proliferating tumor cells were less abundant at tumor front compared to lower grade tumors, while proliferating tumor cells were more abundant in the tumor core. This could indicate an overall shift of proliferation events away from the immediate tumor border and a more disorganized growth pattern.^42^

Two-thirds of the model features selected for grade classification were characteristics of immune cells across all zonal locations. Important grade-associated adaptive immune cell features indicated reduced adaptive immune cell involvement with less differentiation in the tumor: CD4^+^ memory T cells were smaller and less dense in higher grade tumors. Increased memory T cell size is linked to activation and antigen exposure,^43^^,^^44^ suggesting a stronger adaptive immune response in the tumor-adjacent stroma in lower grade OSCC. A similar trend was also observed in tumor-infiltrating CD8^+^ T cells, which were smaller in higher grade tumors. At the tumor front, regulatory CD4^+^ T cells interacted more closely with non-proliferating tumor cells in lower grade compared to higher grade tumors. Regulatory CD4^+^ T cell are recruited to the TIME by effector molecules such as CCL5 and CXCL10,^45^ which are released from proliferating tumor cells.^46^^,^^47^ This finding could point to a close colocalization of regulatory CD4^+^ T cells and proliferating tumor cells; however, further interpretation of this finding is complex and would require more context-dependent information about the function of regulatory T cells, which could be improved in future studies by the inclusion of immune checkpoint markers (e.g., CTLA-4 and PD-1).^48^

In addition to adaptive immune features, several innate immune features differentiated tumors by grade, pointing at functional differences in innate immune cell subsets, specifically in granulocytes. At the tumor front, pMAPKAPK2 activity of granulocytes was dampened in higher grade tumors, possibly reducing proinflammatory cytokine release close to the tumor.^49^ This was another indicator that inflammatory potential might be elevated at the immediate tumor front of lower grade tumors compared to higher grade tumors.^50^ In the stroma, granulocytes expressed increased levels of Ki67 in higher grade tumors. While Ki67 expression is unexpected in terminally differentiated immune cells, expression levels are much lower than in truly proliferating cells such as proliferating tumor cells and could, in the context of cancer-related inflammation, indicate a reactivation of cell cycle marker expression as granulocytes undergo NETosis.^51^^,^^52^

Notably, certain grade-associated features were also correlated with OSCC recurrence and/or mortality, including CD4^+^ T cell size in the stroma, fibroblast distance from the tumor border, and pMAPKAPK2 signaling in granulocytes at the tumor front, suggesting biomarker candidates shared between histological grade and clinical outcomes. Interestingly, the subset of features indicative of recurrence and cancer-related mortality encompassed only immune cell and structural features. In patients without recurrence or cancer-related mortality, fibroblasts were located further from the tumor cell border, and immune cells (e.g., granulocytes) broke through the fibroblast capsule into proximity with the tumor cells. These tumor front granulocytes displayed reduced activation in pMAPKAPK2 signaling in patients with recurrence and cancer-related mortality. This may indicate reduced granulocyte effector functions, as other important proinflammatory signaling proteins, i.e., pERK1/2, pCREB, and pNFκB, were concomitantly suppressed in tumors that later recurred.

### Limitations of the study

We acknowledge certain limitations in this study, including the limited number of patients from two centers, where samples were collected under different circumstances (UOP: incisional biopsies without outcomes data, STA: resection specimens with outcomes data). As the first aim of the research was to create an IMC pipeline encompassing spatial subsetting of OSCC tissue into tissue zones to enable feature standardization and multi-feature class statistical modeling, we focused on histological grade as it is frequently utilized in clinical practice for stratification of tumors. While the WHO grading system correlates with important disease-determining variables, such as lymph node metastasis^7^ and recurrence,^6^ the strength of the WHO system in predicting individual patient outcomes is limited.^28^ Consequently, the biology in the TIME that contributes to recurrence and mortality contains grade-associated and grade-independent features ultimately predictive of clinical outcomes. The analysis of samples from the STA cohort provided an opportunity to validate model features in an independent cohort from a separate center and to identify grade-associated TIME features that also correlated with clinical outcomes. Although several of the model features validated in the second cohort, the multivariable model did not reach statistical significance in this cohort, possibly due to insufficient power or differences in the patient population and type of tissue sections, as well as center-specific differences in sample handling, processing, and storage. As our study was designed and powered for predictive modeling of histological grade, future studies in a larger multicenter patient cohort will be needed to test the generalizability of our findings and their implications for prediction of long-term clinical outcomes. In addition, while the IMC immunoassay contained over 40 antibodies, the list of markers included in our study is not exhaustive. Future studies including additional phenotypic (e.g., for MDSC subsets^53^) or functional markers such as checkpoint molecules, will be particularly informative as inhibitory checkpoint molecules are targetable with current immunotherapies. The association of features with patient HPV-status remained uninvestigated due to limited clinical documentation and limited evidence of association between p16-status and outcomes of OSCC of the tongue.^54^^,^^55^^,^^56^

In summary, the highly multiplexed IMC analysis of the OSCC TIME identified immune correlates, tumor cell characteristics, and structural features accompanying tumor differentiation. We introduce a pipeline for multivariable analysis of highly multiplexed imaging data to address the challenge of integrating spatial information with single-cell data by applying suitable machine learning algorithms. Our spatial subsetting approach increases feature reproducibility and generalizability and facilitates exploitation of the high-resolution content of multiplexed imaging to unravel spatial biology in complex tumor microenvironments with multivariable modeling. Further studies of the OSCC TIME following this model will enhance our understanding of anti-tumor responses and provide important clinical biomarkers to predict outcomes and develop targeted cancer therapeutics.

## STAR★Methods

### Key resources table

**Table:** 

REAGENT or RESOURCE	SOURCE	IDENTIFIER
**Antibodies**		

Purified antibodies, see Table S1	various	various

**Biological samples**		

FFPE tissue blocks	Department of Pathology, Stanford University	NA
FFPE tissue blocks	Arthur A. Dugoni School of Dentistry, University of the Pacific	NA

**Chemicals, peptides, and recombinant proteins**		

PBS	Gibco	Cat#14190250
BSA	Sigma Aldrich	Cat#A3059-50G
Triton™ X-100 (Electrophoresis)	Fisher Scientific	Cat#BP151-100
Xylene	ThermoFisher Scientific	Cat#422680040

**Critical commercial assays**		

Iridium DNA Intercalator	Standard Biotools	Cat#201192B
Target retrieval solution, pH9 (10x)	Agilent	Cat#S2367

**Deposited data**		

Raw and processed data	Zenodo	https://doi.org/10.5281/zenodo.8169946

**Software and algorithms**		

R 4.2.0	https://www.r-project.org	NA
R studio	https://www.rstudio.com/	NA
imcRtools (R package)	Windhager et al. 2021^32^	10.18129/B9.bioc.imcRtools
RPhenograph (R package)	Levine et al. 2015^30^	https://github.com/JinmiaoChenLab/Rphenograph
Python 3.9.0	https://www.python.org/	NA
Stabl (Python package)	Hedou et al.^39^	https://github.com/gregbellan/Stabl
Steinbock (Docker version)	Windhager et al. 2021^32^	https://github.com/BodenmillerGroup/steinbock
Ilastik 1.4	https://www.ilastik.org/	NA
Cellprofiler 4.2.5	https://cellprofiler.org/	NA

**Other**		

Helios mass cytometer	Standard Biotools	NA
Hyperion imaging system	Standard Biotools	NA

### Resource availability

#### Lead contact

Further information and requests for resources and reagents should be directed to and will be fulfilled by the lead contact, Brice Gaudillière (gbrice@stanford.edu).

#### Materials availability

All materials used in this study are commercially available, as specified in the key resources table and Table S1. This study did not generate new unique reagents.

#### Data and code availability

Supplemental figures and tables, raw data, processed data, and source code required to reproduce analysis steps and figures are publicly available on Zenodo (https://doi.org/10.5281/zenodo.8169946). All data needed to evaluate the conclusions in the paper are present in the paper or the supplemental information. Any additional information required to reanalyze the data reported in this paper is available from the lead contact upon request.

### Experimental model and study participant details

#### Study design

Using highly multiplexed IMC, we analyzed the organizational and architectural patterns in the TIME in OSCC to identify features indicative of OSCC tumor grade in a biorepository of 24 treatment-naïve OSCC biopsies. In an independent biorepository of 24 treatment-naïve OSCC resection samples, we validated the model’s features and investigated their association with clinical outcomes in a post-hoc analysis. While ROI selection in both biorepositories consistently focused on the tumor-stroma interface, the two cohorts were collected at different institutions and had circumstantial differences: The first cohort comprised incisional biopsies of invasive OSCC from 24 patients that were obtained between 2009 and 2011 at UOP, San Francisco, CA (IRB2021-161). Available patient information included race, ethnicity, sex, patient age, and histological grade (Table S2), but not clinical outcomes as patients were referred to other centers for treatment following OSCC diagnosis. The second cohort, originating from larger resection specimens of primary, treatment-naïve resection cases of OSCC, was collected at Stanford Hospital (IRB-63024). Here, in addition to patient demographics, adjuvant treatment modality and clinical outcomes, including recurrence within three years and cancer-related mortality within five years of resection with clear margins, were available (Tables S2 and S3). The unique design of this study allowed for comparisons of intra- and interpatient heterogeneity between biopsy and resection samples, independent validation of the model features, and a post-hoc analysis for feature association with outcome.

#### Biological materials

For the UOP cohort, three ROIs (1000 × 500 μm) were preselected on representative H&E sections of the FFPE-embedded tumor biopsies. For one biopsy, the tissue dimensions of the biopsy allowed for only two ROIs. For the Stanford cohort, a tissue microarray (1.5 mm cores) was prepared from FFPE samples of 24 patients treated at the Stanford University for primary resection of treatment-naïve OSCC. The TMA was composed of samples from 23 patients with three cores and one patient with two cores. Per core, an ROI of 1000 × 500 μm was ablated.

### Method details

#### Antibody validation

Antibodies used in this investigation are listed in Table S1. When ordered as carrier-free purified aliquots, antibodies were validated by immunohistochemistry on available biopsies of OSCC to confirm adequate staining intensity, optimal signal-to-noise ratio, and expected staining patterns with a clinical pathologist. Antibodies were stored at concentrations of 0.5 μg/mL at 4°C. To reduce channel overlap, which is minimal for the IMC platform and predominantly occurs between adjacent channels, placement of low intensity markers next to high intensity markers was avoided during the panel design. Conjugation of antibodies with metal tags was performed following the manufacturer’s instructions. Titration of the antibody panel was performed to identify the optimal titer for each antibody.

#### Tissue staining

Each cohort was processed in one batch. Tissues were sectioned to a thickness of 4 μm from the FFPE blocks and baked at 70°C followed by deparaffinization and rehydration with sequential washes in xylene (2x), ethanol (100%, 95%, 80%, 70%, 1x each), and ddH_2_O. Antigen retrieval was achieved using a Target Retrieval Solution (pH9, DAKO) during an incubation of 30 min at 96°C. Non-specific binding sites were blocked with 3% BSA at room temperature for 45 min and then stained for 16 h overnight at 4°C in a humidity chamber with the antibody cocktail in 1% BSA filtered through a 0.1 μμm centrifugal filter. To reduce batch effects and guarantee intraindividual comparability of results, the antibody cocktail was produced in one master mix for all samples and the staining area was normalized to a reproducible square of 5 × 5 mm when encircled with a pap pen. Slides were washed with ddH_2_O, PBS, and 0.1% Triton X-, then incubated with an intercalator (Ir^192^ and Ir^193^) for 8 min. The slides were then washed with ddH_2_O and left to dry.

#### Imaging mass cytometry data acquisition

IMC images were acquired using a Hyperion imaging system (Standard Biotools) connected to a Helios mass cytometer (Standard Biotools) according to the manufacturer’s instructions. With daily calibration, the tissue slides were laser-ablated at a resolution of ∼1 μm (ablation energy: 2dB) and a frequency of 200 Hz. A total of 142 ROIs was acquired (71 ROIs per cohort) and stored as individual MCD and.txt files.

#### Imaging mass cytometry analysis

##### Cell segmentation

Cells were segmented using the segmentation pipeline described in Windhager et al.^33^ and the Mesmer algorithm was applied to each ROI.^26^ Before segmentation, the images were processed to remove hot pixels. Three channels were then pooled to represent the nuclei: histone H3, Ir191, and Ir193. For the cell membrane, the signal of 24 channels was summed to generate one single cell membrane marker (Table S1). Cells were identified as primary objects and single object data were exported as mean marker expression in each cell for each channel.

##### Cell type identification

The mean channel intensities per cell were Arcsinh transformed (cofactor = 1) to rescale the expression levels across the cohort and to deal with varying levels of background signal. The transformed channel intensities were used for the initial classification step employing the PhenoGraph clustering algorithm^27^ embedded in the IMC data analysis workflow by Windhager et al.^33^ to identify the 30 nearest neighbors. In this step, cells were clustered by the expression of 12 major lineage markers (aSMA, CD3, CD4, CD8, CD14, CD20, CD31, CD45, CD68, collagen, pancytokeratin, and vimentin). The produced cell clusters were then combined into seven major cell subsets based on biologically coherent marker expression patterns. For further subclassification of cell subsets, the respective major cell populations were clustered into highly granular subclusters using PhenoGraph: CD4^+^ T cells were clustered by expression of FoxP3 and CD45RA, myeloid cells by the expression of CD11b, CD11c, CD14, CD16, CD68, CD163, CD206, and CD209, and tumor cells by the expression of Ki67 (Table S1). The produced subclusters were then combined into biologically relevant subpopulations of CD4^+^ T cells (regulatory = FoxP3^+^, naive = CD45RA^+^, memory = CD45RA^−-^), tumor cells (proliferating = Ki67^+^, non-proliferating = Ki67^-^), and myeloid cells (M1 macrophages = CD68^+^CD206^-^, M2 macrophages = CD68^+^CD163^+^CD206^+^, monocytes = CD14^+^CD68^low^, granulocytes = CD14^low^CD11b^+^Granzyme B^+^, dendritic cells = CD11c^+^, M-MDSCs = CD14^+^CD11b^+^).

##### Tumor masks and zonal area calculation

Tumor masks were generated using Ilastik. A pixel classifier was trained on a subset of ten images per cohort to produce probability maps of tumor and stroma. Using a customized cellprofiler pipeline, two-color masks were generated for the tumor area A_r_, an expanded tumor area A_e_ (expansion of 20 pixels from the tumor border) and a shrunken tumor area A_s_ (shrinking of 20 pixels from the tumor border). Each acquired image contained 500,000 pixels at a resolution of one pixel per μm^2^. The stromal zone area was calculated as the A_ROI_ - A_e_, the tumor front zone area as A_e_ - A_s_, and A_s_ represented the tumor core zone area.

##### Neighborhood and cell-cell interaction quantification

For each individual cell, the five nearest cells were regarded as neighboring if their distance apart did not exceed 20 μm (Euclidean distance from the cell border). Neighborhood coefficients were calculated and averaged within cell subsets.

### Quantification and statistical analysis

#### Dataset assembly and feature classes

The generated IMC data were stringently organized into a high-dimensional dataset with four feature classes, including zonal cell densities (45 features), cell-type specific functional activities (420 features), single-cell spatial metavariables (74 features), and cell-cell interaction features (374 features). All four feature classes were reported per respective tissue zone as described above. To avoid distortion of subsequent features (i.e., function, spatial metavariables, and cell-cell interaction) solely based on imbalances of cell abundance between lower and higher grade, cell populations with a median zonal cell density of zero were excluded from all other feature classes describing this respective zone. The second class represented the functional features of the 14 markers used in the study. To compute these features, we extracted the mean expression across each cell population (420 features). These markers included eight phosphorylated signaling proteins (pp38, pSTAT1, pSTAT3, pCREB, pNFκB, pERK1/2, pMAPKAPK2, pS6), intracellular proteins (VEGF, Granzyme B, Ki67, E-Cadherin), and surface markers (CD36, CD44). The third feature class included three single-cell spatial metavariables: cell size, cell eccentricity, and cell distance from the tumor border, extracted as a mean for each cell type (74 features). While cell size and eccentricity were comparable throughout the dataset, cell distance varied due to different tumor-stroma area ratios on the images and was, therefore, only considered for the tumor front zone. Finally, spatial cell-cell interactions were calculated as neighborhood coefficients of the five nearest neighbors per individual cell based on Euclidean object border distances within a threshold of 20 μm. The mean neighborhood coefficient per cell subset was included as a fourth features class (374 features) to complete the full dataset of 913 features per sample.

#### Analysis of intrasample heterogeneity

The study outline of two independent cohorts with different sample collection modalities (tumor incisional biopsy vs. larger resection specimen) allowed us to compare intrapatient, interpatient, and inter-cohort heterogeneity within and between the two cohorts. As an estimation for the observed variability, we calculated CVs for the cell densities of all cell populations before and after spatial subsetting. Intrapatient CVs were calculated as CV across the three ROIs from one respective patient and interpatient CVs across all patients within one cohort by randomly selecting one ROI per patient.

#### Multivariable modeling approach

Our multivariable approach to the UOP IMC dataset was based on a machine learning method called Stabl that implements iterative sparse learner (in this case LASSO) models in a rigorous bootstrapping and cross-validation procedure adapted from stability selection.^34^^,^^35^ Model optimization was achieved using a 5-fold Monte Carlo cross-validation strategy. Within each cross-validation fold, LASSO models were built on each feature class individually across 500 bootstrapping iterations subsampling 50% of the training data (Figure S8). For a matrix X of features from a given feature class of N samples and a vector of grade Y, the LASSO algorithm calculates coefficients ***β*** to minimize the error term *L*(***β***) = ||*Y*−*X* ***β***||2+***λ***||***β***||1. Using L1 regularization increased the model sparsity, meaning more feature coefficients are set to zero, thereby limiting the number of features in the final model. This, in combination with the bootstrapping procedure, strengthens the biological interpretability and model robustness. At each iteration, features with non-zero coefficients were considered as selected by the LASSO procedure on the bootstrapped dataset. Features from all four feature classes that were selected in more than 50% of the iterations were selected for the final model. Integrating the most informative features of all features classes, a logistic regression was built, and its performance was evaluated on the samples held out for testing.

#### Univariate analysis and association with outcome

Statistical comparisons were performed on ROI level, assuming independence between single images. Given the heterogeneity of tumors with respect to cell types and marker expression, this assumption was considered reasonable and appropriate to describe the local tissue properties. Univariate analysis restricted to the selected model features was performed using the stats package in the R environment (http://www.r-project.org/). Statistical significance of the model features was assessed using a two-sided Mann Whitney test for stratification of tumor grade, tumor recurrence within three years, and cancer-related mortality within five years of resection with clear margins.
