# Supplementary material for: Spatial subsetting enables integrative modeling of oral squamous cell carcinoma multiplex imaging data
Source: iScience. 2023 Nov 20;26(12):108486. doi: 10.1016/j.isci.2023.108486 (PMC10730356; doi:10.1016/j.isci.2023.108486)
Supplement: Document S1. Figures S1–S11 and Tables S1–S3 [file mmc1.pdf]

iScience, Volume *26*

Supplemental information

Spatial subsetting enables integrative modeling of oral squamous cell carcinoma multiplex imaging data

Jakob Einhaus, Dyani K. Gaudilliere, Julien Hedou, Dorien Feyaerts, Michael G. Ozawa, Masaki Sato, Edward A. Ganio, Amy S. Tsai, Ina A. Stelzer, Karl C. Bruckman, Jonas N. Amar, Maximilian Sabayev, Thomas A. Bonham, Joshua Gillard, Maïgane Diop, Amelie Cambriel, Zala N. Mihalic, Tulio Valdez, Stanley Y. Liu, Leticia Feirrera, David K. Lam, John B. Sunwoo, Christian M. Schürch, Brice Gaudilliere, and Xiaoyuan Han

Supplemental material

Supplemental Figures

S1 Cell composition of the TIME in OSCC (STA).
S2 Overview of all cell masks colored by cell type (UOP).
S3 Overview of all cell masks colored by cell type (STA).
S4 Cell densities across samples and tissue zones (UOP).
S5 Cell densities across samples and tissue zones (STA).
S6 Intrapatient test-retest reliability between ROIs of one individual patient per cell type.
S7 Intrapatient and interpatient variability of cell population densities comparing UOP and STA.
S8 Flowchart of cross-validation and bootstrapping procedure.
S9 Univariate comparative analysis of model features between higher and lower grade (UOP).
S10 Univariate comparative analysis of model features between higher and lower grade (STA).
S11 Subset analysis of model features between well, moderately, and poorly differentiated tumors (STA).

Supplemental Tables

S1 Antibody panel.
S2 Cohort characteristics for UOP and STA cohort.
S3 Adjuvant treatment and patient outcomes in the STA cohort.

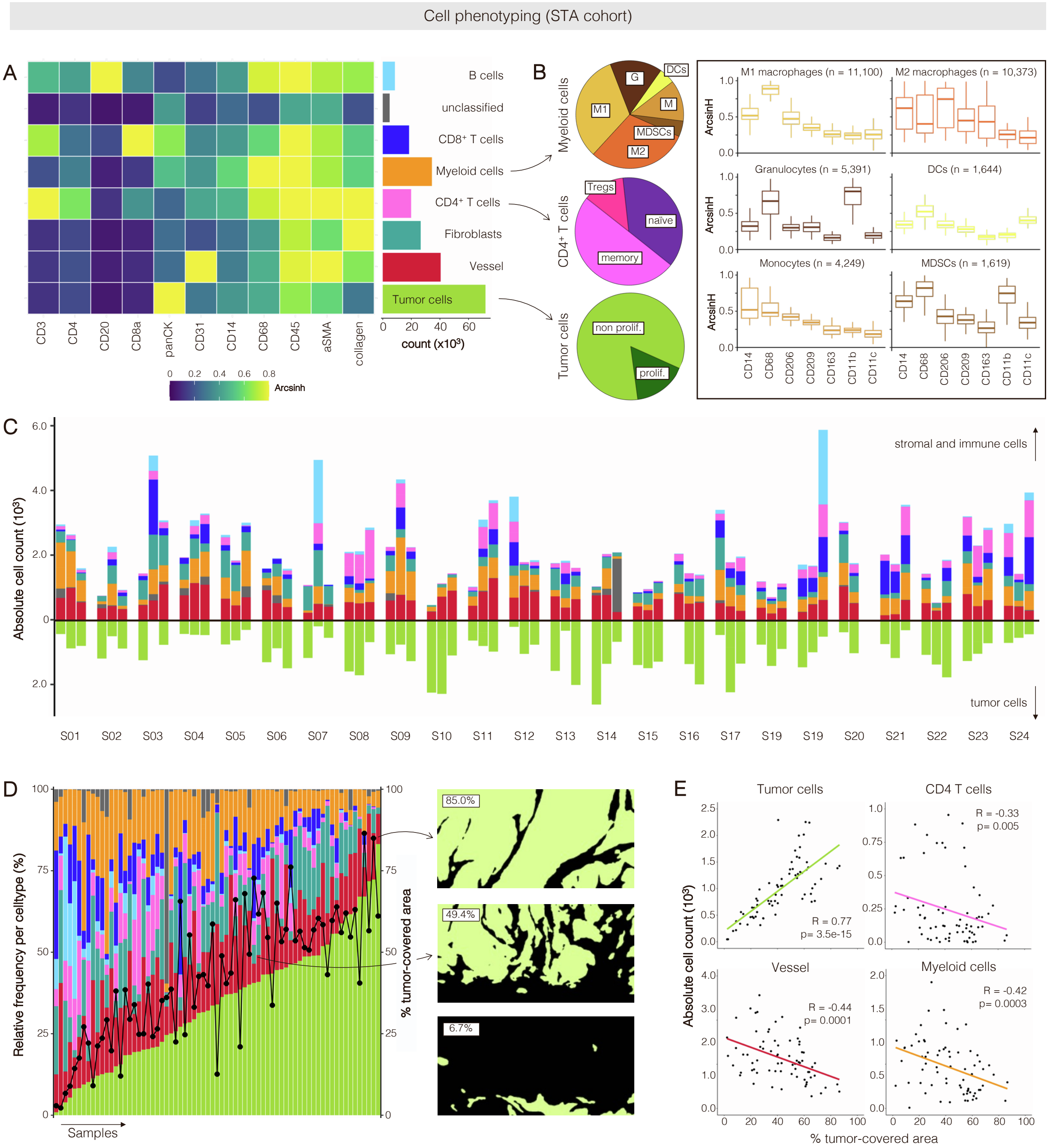

Figure S1. Cell composition of the TIME in OSCC (STA), Related to Figure 2. A Heatmap of mean marker expression levels and absolute count of major cell subsets. **B** Relative distribution of myeloid (top left), CD4⁺ T cell (middle left), and tumor cell (bottom left) subpopulations. Marker expression levels of the respective myeloid subpopulations (right). Boxplots depict median and IQR. **C** Inter- and intrapatient variability in cell abundance across ROIs. Absolute cell counts per main cell subset are depicted. **D** Relative frequency (cell count per total cell count) per main cell subset for all samples ordered by increasing tumor cell frequency (left). Black dots represent the proportion of tumor-covered area per ROI. Examples of tumor masks varying in tumor-covered area (right panels). **E** Spearman correlation between absolute cell count and the proportion of tumor-covered area per sample.

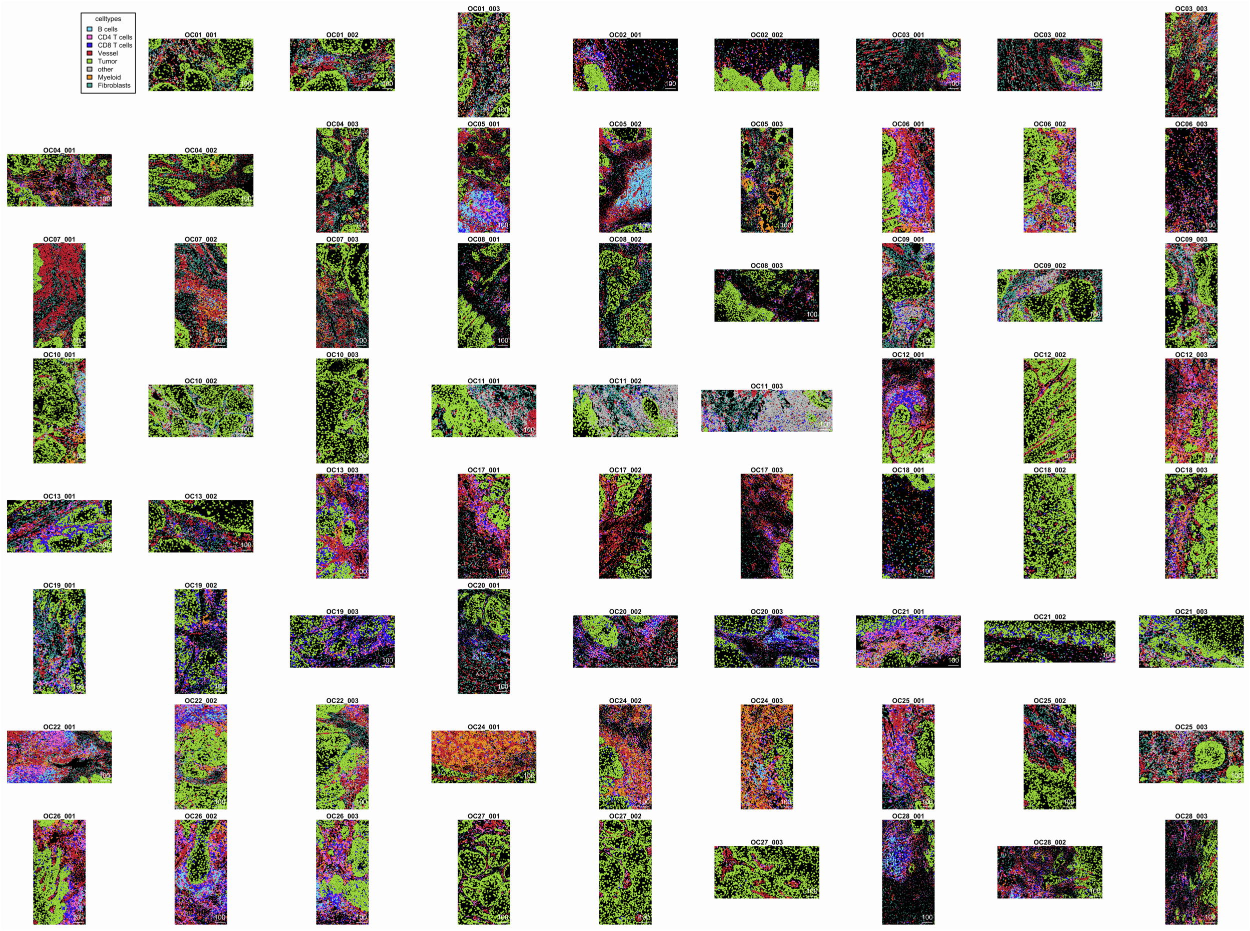

Figure S2. Overview of all cell masks colored by cell type (UOP), Related to Figure 2. Main cell types (tumor cells, vessel, fibroblasts, CD4 T cells, CD8 T cells, B cells, myeloid cells, other) are depicted.

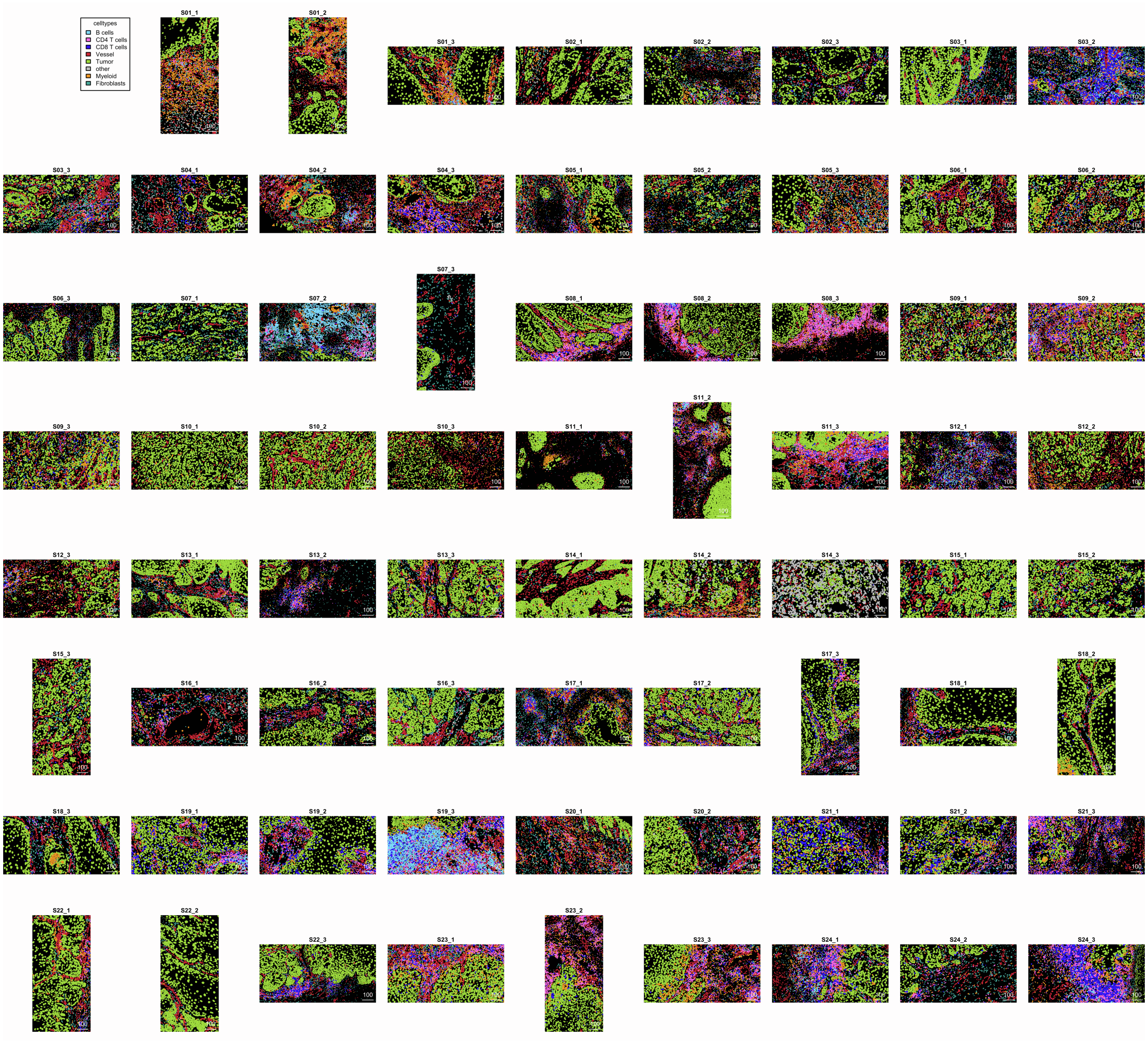

Figure S3. Overview of all cell masks colored by cell type (STA), Related to Figure 2. Main cell types (tumor cells, vessel, fibroblasts, CD4 T cells, CD8 T cells, B cells, myeloid cells, other) are depicted.

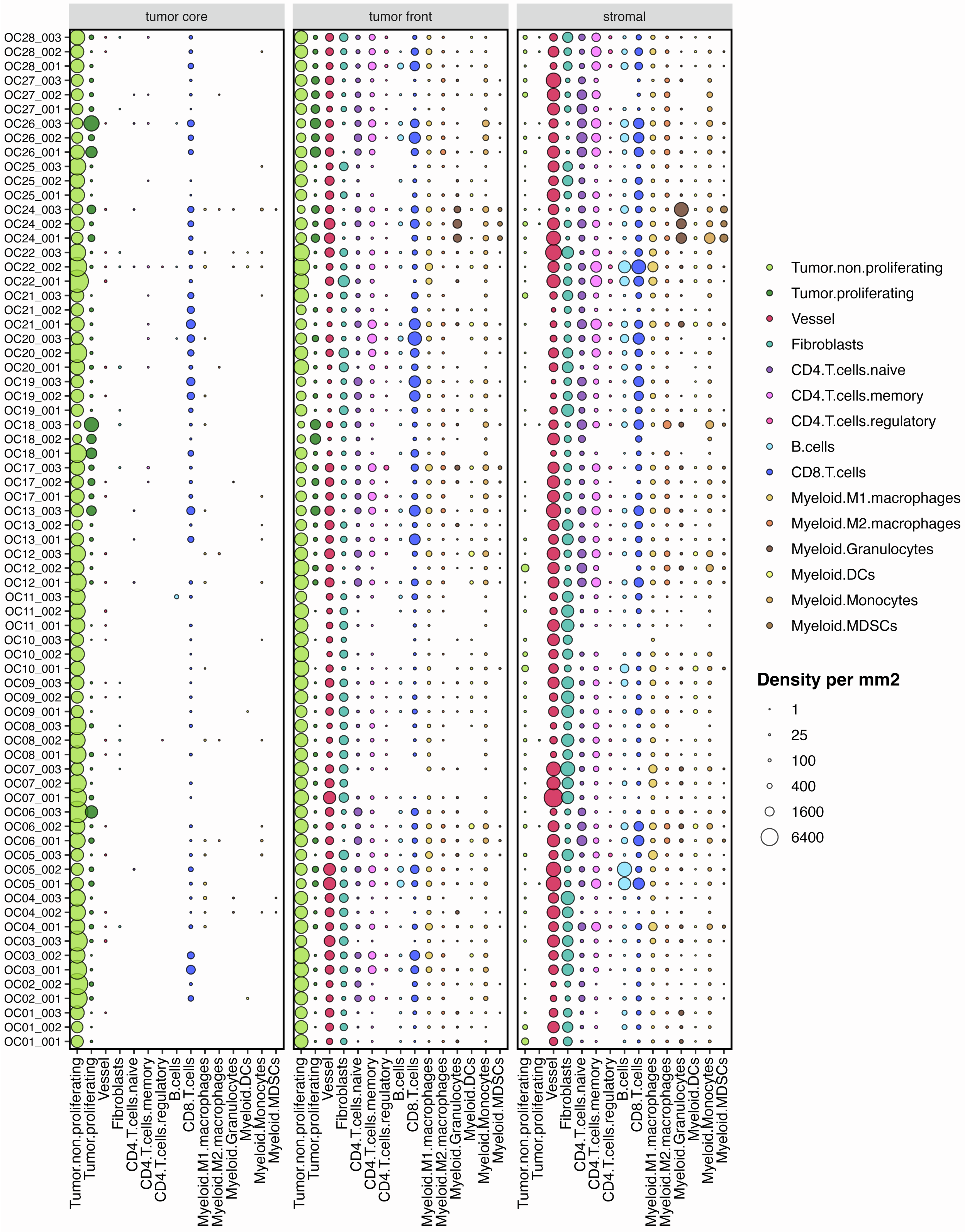

Figure S4. Cell densities across samples and tissue zones (UOP), Related to Figure 3. Node size indicates the zonal cell density after subsetting (tumor core, tumor front, stromal) for all 15 identified cell subpopulations across all samples.

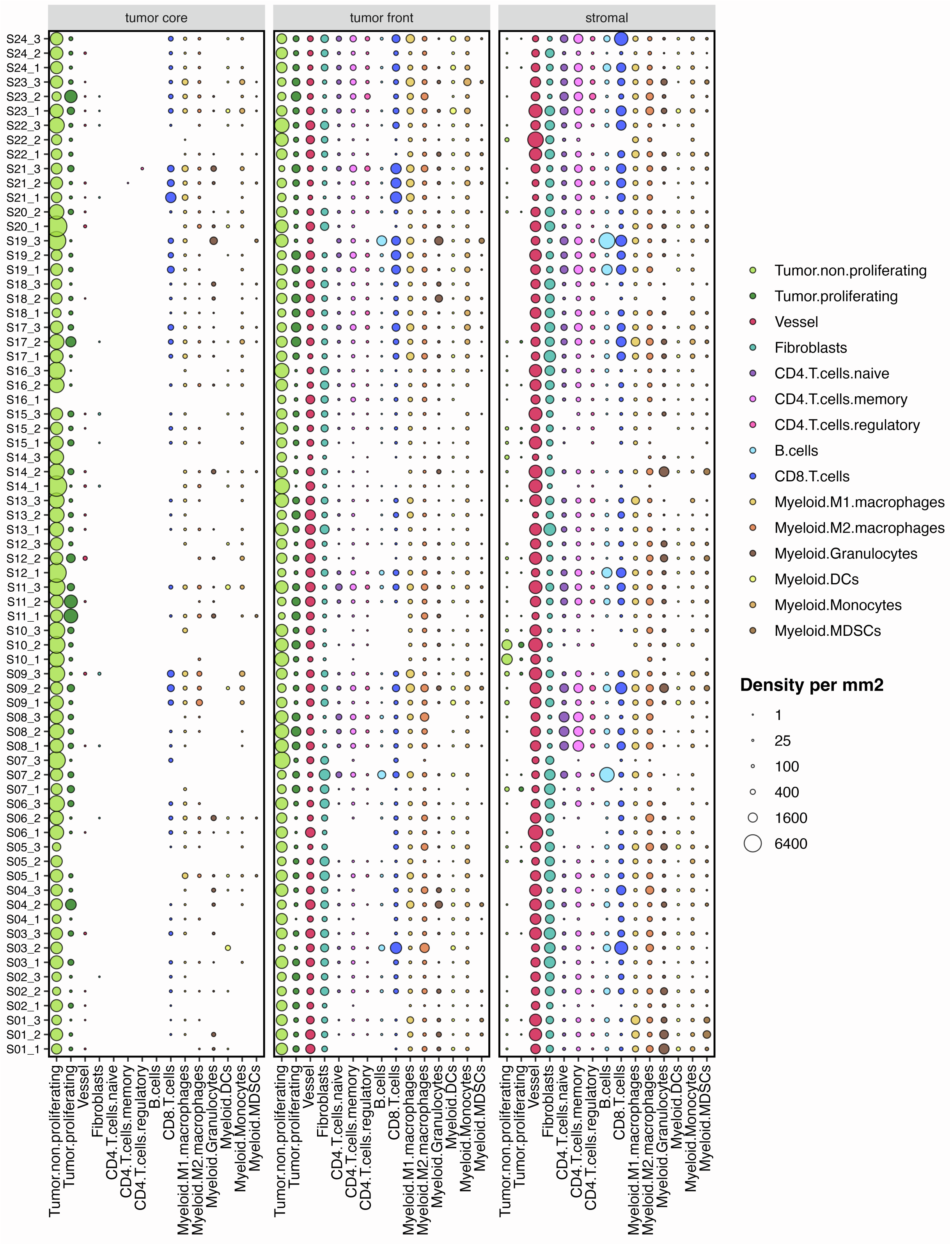

Figure S5. Cell densities across samples and tissue zones (STA), Related to Figure 3. Node size indicates the zonal cell density after subsetting (tumor core, tumor front, stromal) for all 15 identified cell subpopulations across all samples.

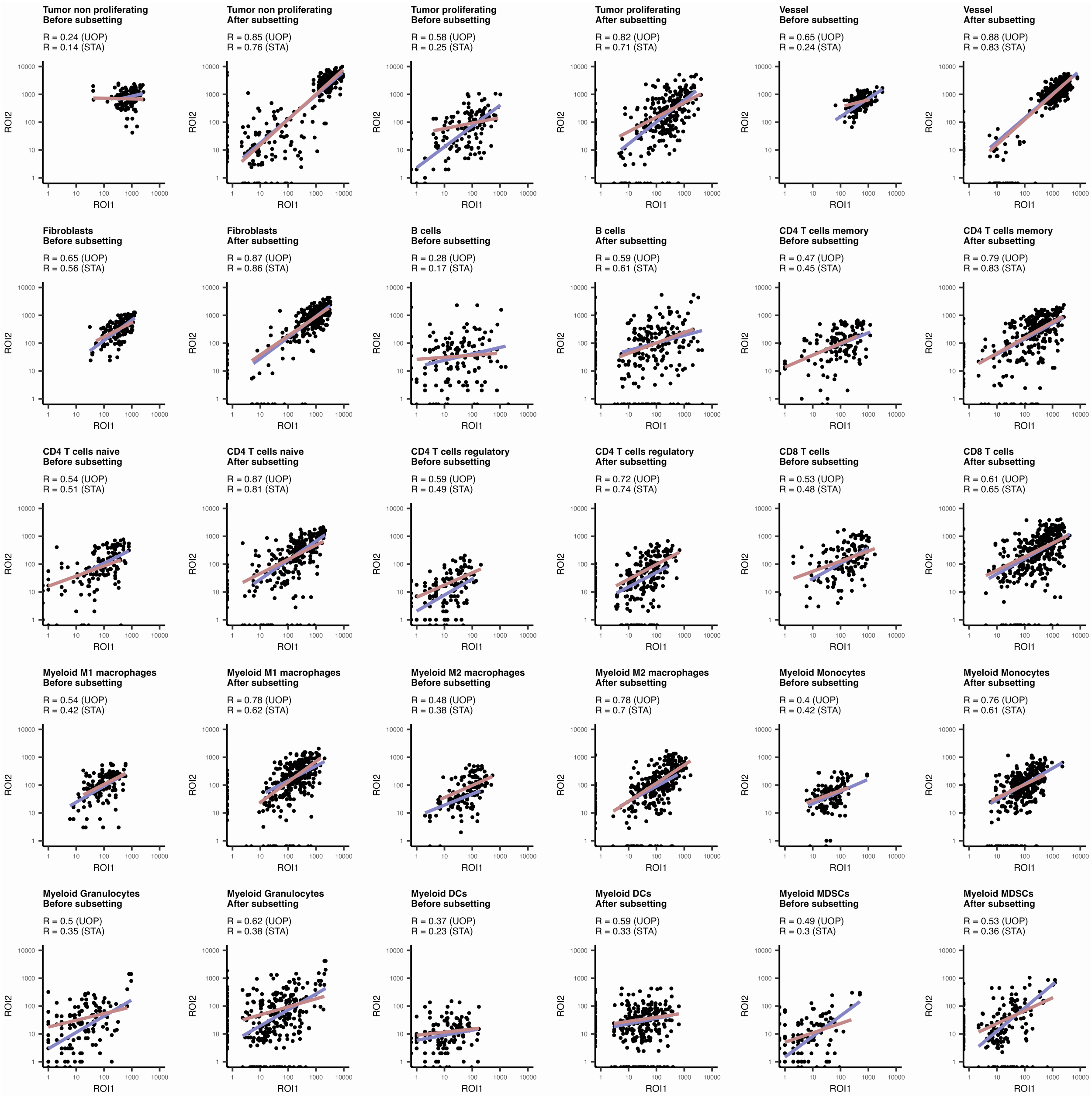

Figure S6. Intrapatient reproducibility between ROIs of the same patient per cell type before and after spatial subsetting, Related to Figure 4. Pairwise comparison of agreement between ROIs of the same patient for quantification of each cell type. Before spatial subsetting, the absolute count per cell population is compared between all possible pairwise combinations of two ROIs for each individual patient. After spatial subsetting, the cell population density per cell population is compared between all possible pairwise combinations of two ROIs for each individual patient. Correlation is reported for both cohorts separately (UOP: blue, STA: maroon) and was calculated using a Spearman correlation.

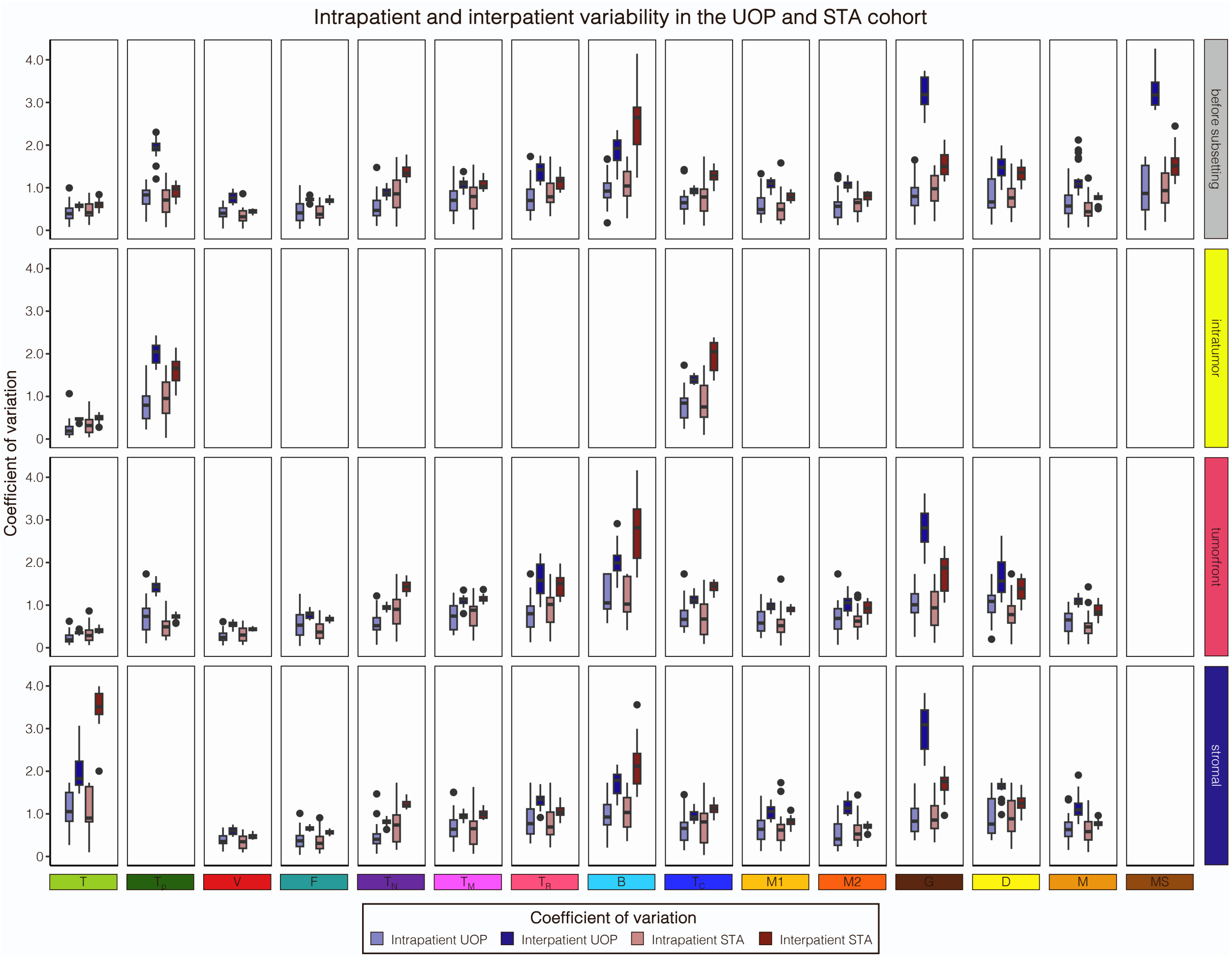

Figure S7. Intrapatient and interpatient variability of cell population densities comparing UOP and STA, Related to Figure 4. Intrapatient and interpatient coefficients of variation (CVs) of all cell population densities before and after spatial subsetting in the two cohorts (UOP: blue, STA: maroon). CVs after spatial subsetting were only calculated for cell populations with a non-zero absolute median. For intrapatient CVs, boxplots depict medians and IQR of 24 intrapatient CVs calculated for each patient independently. For interpatient CVs, boxplots depict medians and IQR of 24 bootstrap iterations randomly sampling one image per patient.

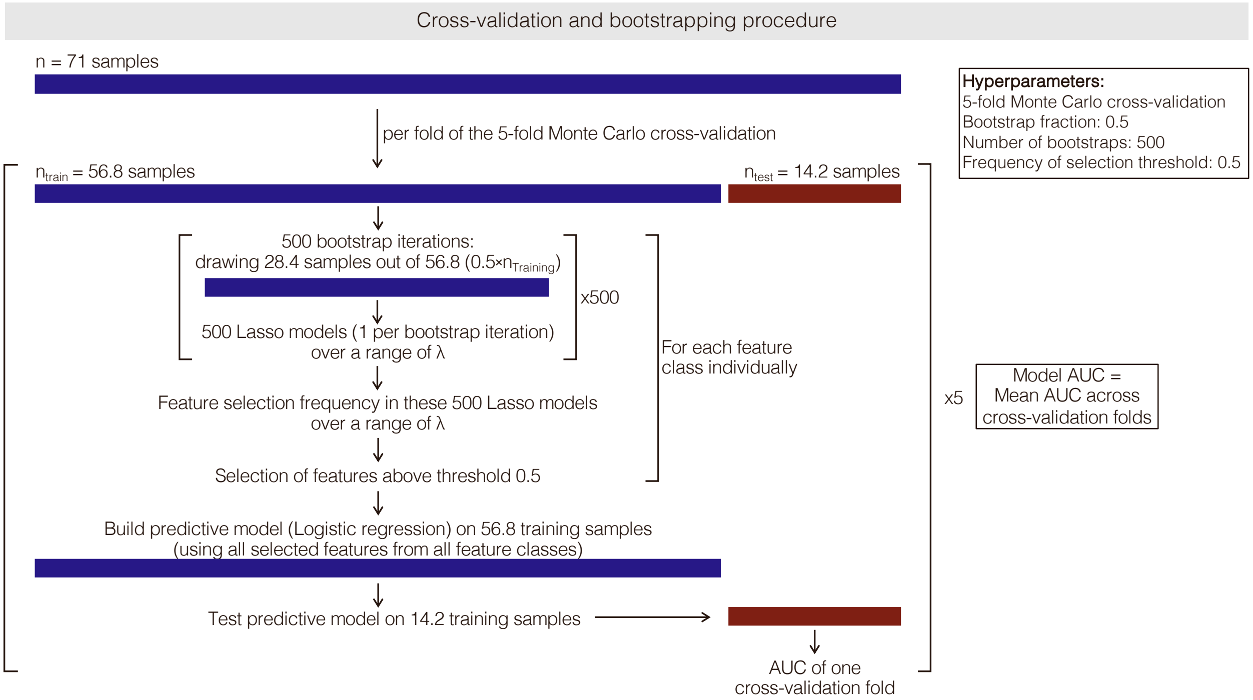

Figure S8. Model cross-validation and bootstrapping procedure, Related to Figure 5. Flowchart depicting the employed cross-validation and bootstrapping procedure.

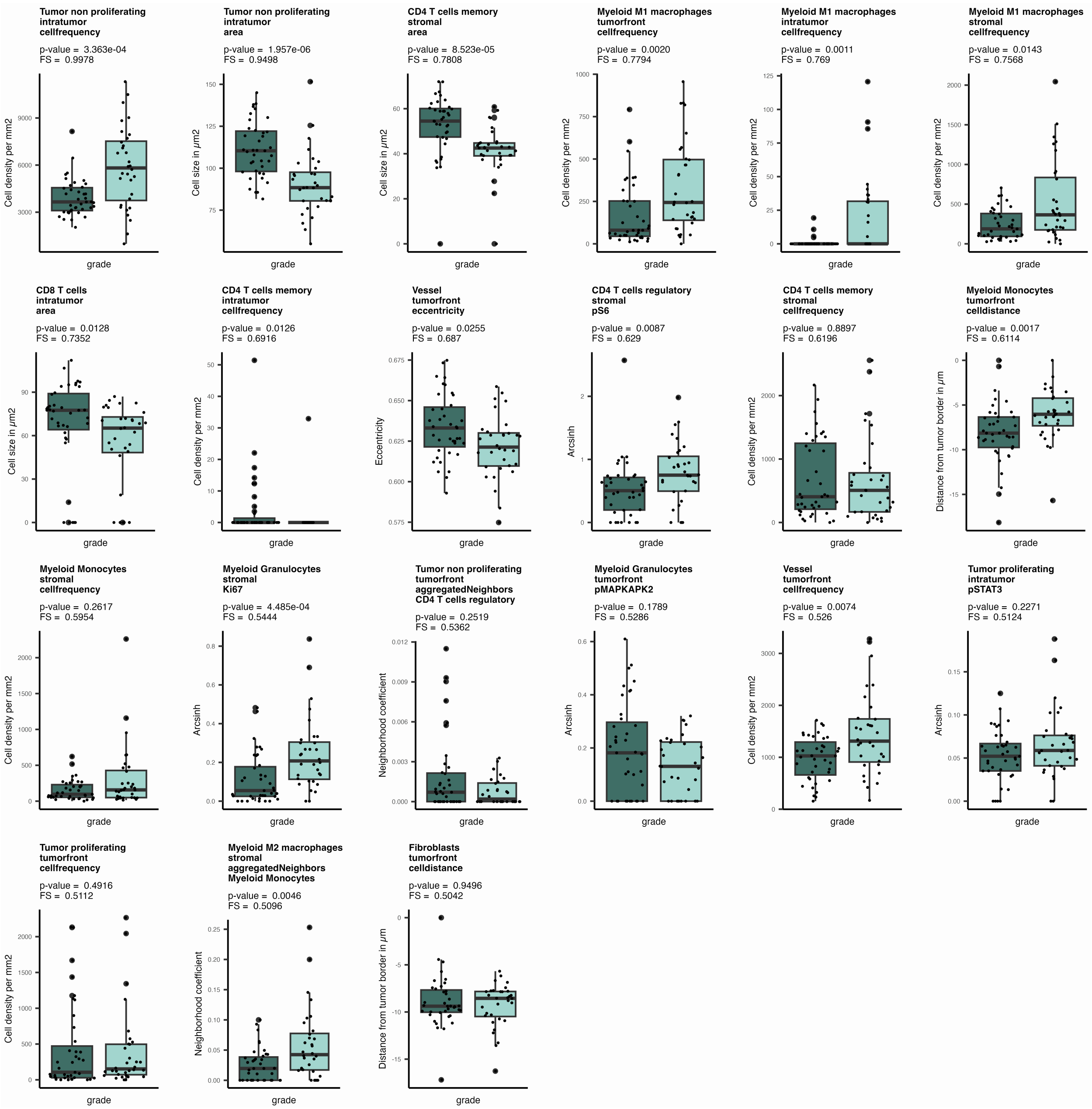

Figure S9. Univariate comparative analysis of model features between higher and lower grade (UOP), Related to Figure 5. Significance between grades (lower grade: dark turquoise, higher grade: light turquoise) was calculated using a Mann Whitney U-test. Features are ordered in descending order of frequency of selection (FS) across all bootstrap model iterations. Boxplots depict medians and IQR.

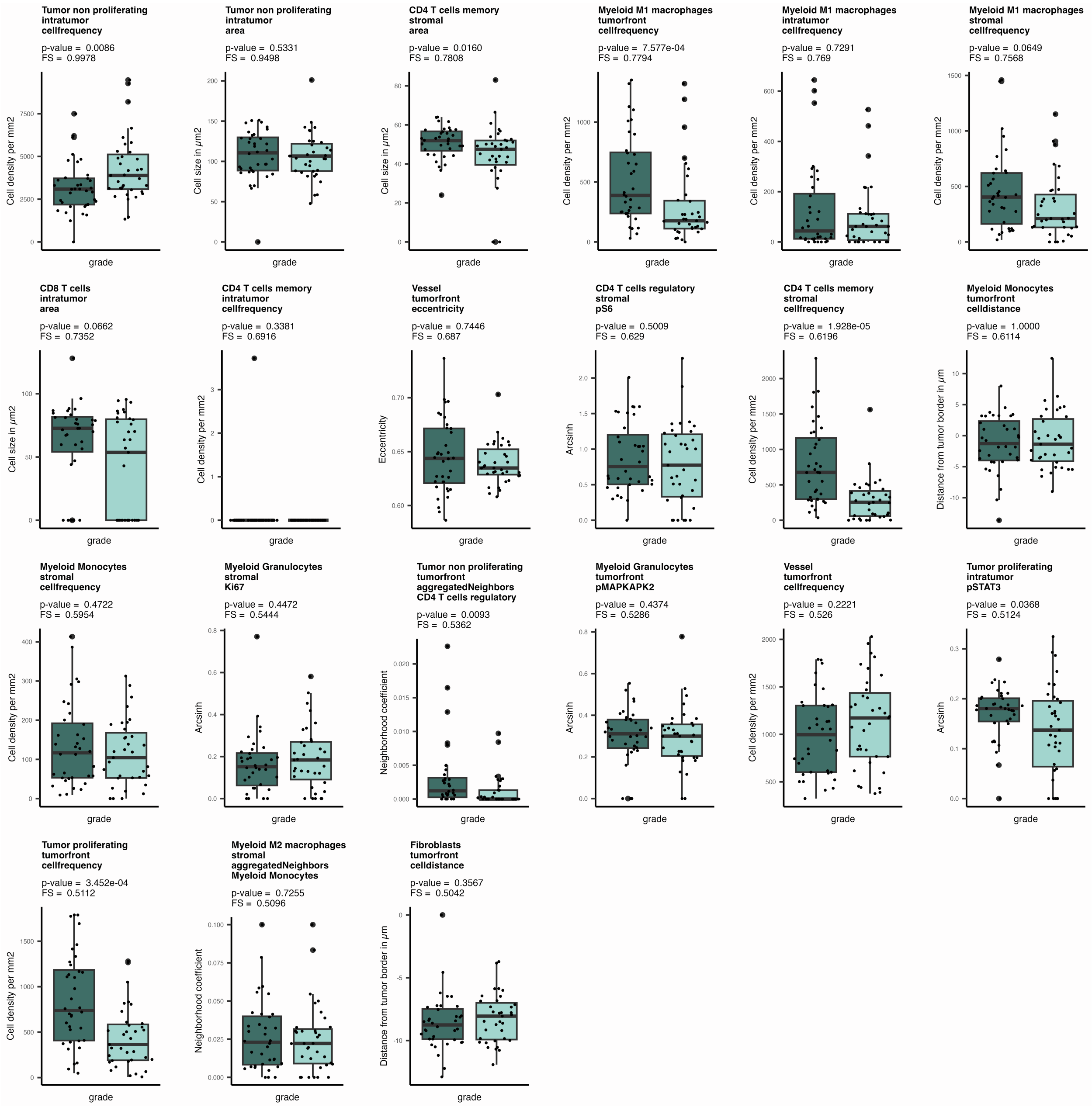

Figure S10. Univariate comparative analysis of model features between higher and lower grade (STA), Related to Figure 5. Significance between grades (lower grade: dark turquoise, higher grade: light turquoise) was calculated using a Mann Whitney U-test. Features are ordered in descending order of frequency of selection (FS) across all bootstrap model iterations. Boxplots depict medians and IQR.

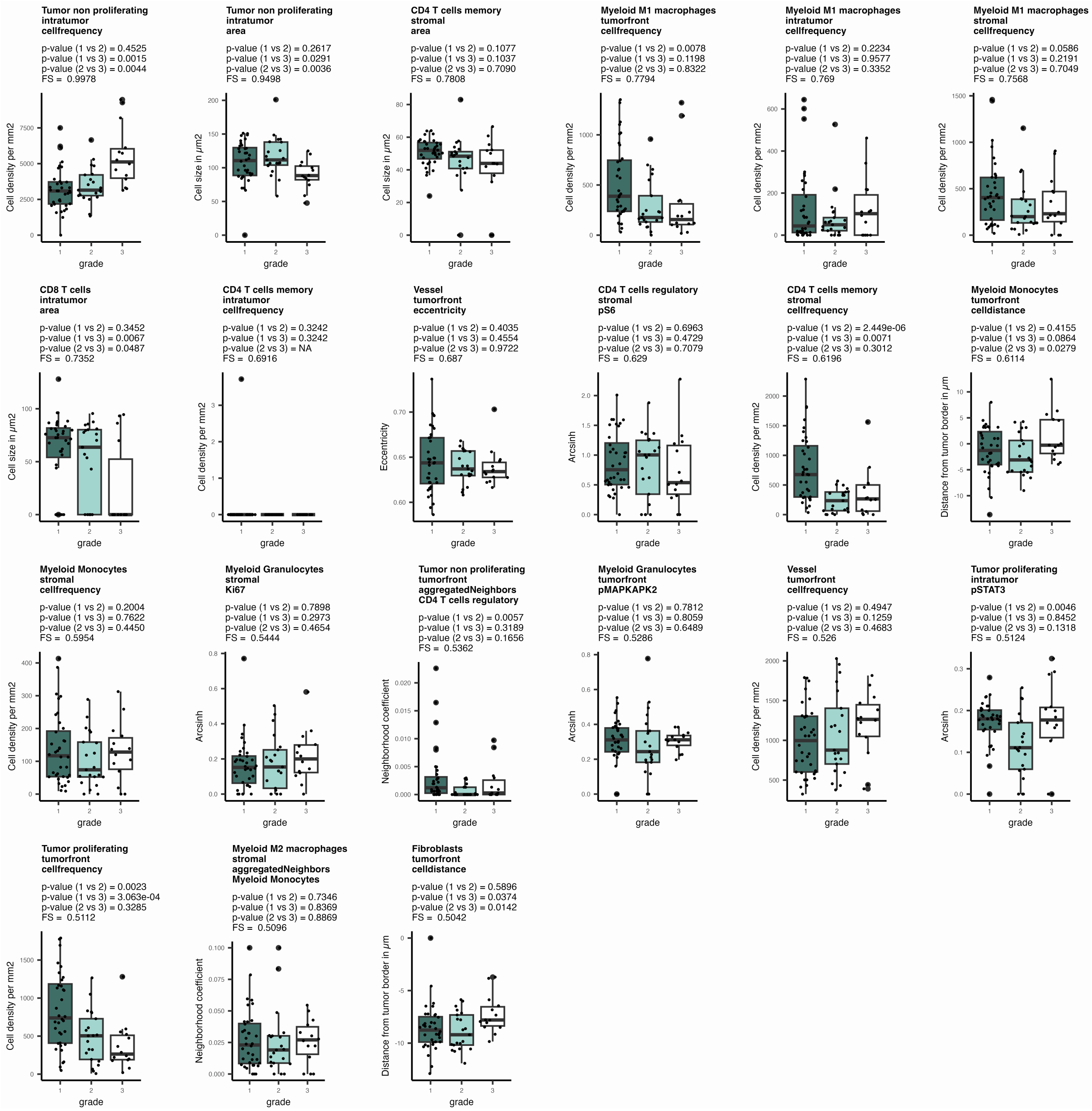

Figure S11. Subset analysis of model features between well, moderately, and poorly differentiated tumors (STA), Related to Figure 5. Significance between grades well (grade 1), moderately (grade 2), and poorly (grade 3) differentiated tumors was calculated using a Mann Whitney U-test. Features are ordered in descending order of frequency of selection (FS) across all bootstrap model iterations. Boxplots depict medians and IQR.

Metal Tag	Target	Antibody Clone	Vendor	Dilution	c (µg/ml)	Segmentation	Coarse	Fine	UOP	STA
In115	Podoplanin	D2-40	Biolegend	100	5	membrane			x	
In115	CD15	W6D3	Fluidigm	100	5					x
Ce140	Vimentin	D21H3	CST	200	2.5	membrane	yes		x	x
Pr141	aSMA	1A4	Invitrogen	100	5	membrane	yes		x	x
Nd142	CD206	E2L9N	CST	50	10	membrane		Myeloid cells	x	x
Nd143	CD45RA	HI100	Biolegend	200	2.5	membrane		CD4 T cells	x	x
Nd144	CD14	EPR3653	Abcam	300	1.67	membrane	yes		x	x
Nd145	CD209	DCN46	BD Bioscience	50	10	membrane		Myeloid cells	x	x
Nd146	CD16	EPR16784	Abcam	200	2.5	membrane			x	x
Sm147	CD163	EDHu-1	Fluidigm	100	5	membrane		Myeloid cells	x	x
Nd148	PanCK	AE1/AE3	Biolegend	200	2.5	membrane	yes		x	x
Sm149	CD11b	EP1345Y	Abcam	100	5	membrane		Myeloid cells	x	x
Nd150	CD86	37711	Invitrogen	100	5	membrane			x	
Nd150	HLADR	EPR3692	Abcam	100	5	membrane				x
Eu151	pp38	36/p38	BD Bioscience	50	10				x	x
Sm152	CD45	D9M8I	CST	200	2.5	membrane	yes		x	x
Eu153	pSTAT1	14/p-STAT1	BD Bioscience	50	10				x	x
Sm154	pSTAT3	M9C6	CST	50	10				x	x
Gd155	FOXP3	236A/E7	Abcam	100	5			CD4 T cells	x	x
Gd156	CD4	EPR6855	Abcam	100	5	membrane	yes		x	x
Gd157	CD36	D8L9T	CST	100	5	membrane			x	x
Gd158	E-Cadherin	24E10	CST	100	5	membrane			x	x
Tb159	CD68	KP1	Biolegend	200	2.5	membrane	yes	Myeloid cells	x	x
Gd160	CD31	EP3095	Abcam	200	2.5	membrane	yes		x	x
Dy161	CD20	H1	Fluidigm	100	5	membrane	yes		x	x
Dy162	CD8a	C8/144B	Biolegend	100	5	membrane	yes		x	x
Dy163	VEGF	G153-694	Fluidigm	200	2.5				x	x
Dy164	CD3	D7A6E	CST	100	5	membrane	yes		x	x
Ho165	pCREB	87G3	CST	100	5				x	x
Er166	pNFkB	EPR17622	BD Bioscience	100	5				x	x
Er167	Granzyme B	EPR20129-217	Fluidigm	100	5				x	x
Er168	Ki67	B56	Fluidigm	100	5			Tumor	x	x
Tm169	Collagen	Polyclonal	Fluidigm	200	2.5		yes		x	x
Er170	CD44	IM-7	Biolegend	100	5	membrane			x	x
Yb171	pERK1/2	D13.14.4E	CST	100	5				x	x
Yb172	CD11c	EP1347Y	Abcam	100	5	membrane		Myeloid cells	x	x
Yb173	CD56	EPR2566	Abcam	300	1.67	membrane			x	x
Yb174	pMAPKAPK2	27B7	CST	100	5				x	x
Lu175	pS6	N7-548	CST	100	5				x	x
Yb176	Histone H3	D1H2	Fluidigm	300	1.67	nuclear			x	x
Ir191	DNA	Iridium	Fluidigm	1000	0.5	nuclear			x	x
Ir193	DNA	Iridium	Fluidigm	1000	0.5	nuclear			x	x

Table S1. Antibody panel, Related to Figure 1. Columns coarse and fine indicate whether markers were used for RPhenograph clustering of main cell types (coarse) or subpopulations (fine). c = concentration.

		Full Cohort	Well differentiated	Moderately differentiated	Poorly differentiated
**UOP Cohort**		n = 24	n = 13	n = 10	n = 1
Sex	Female	11 (46%)	7 (54%)	4 (40%)	0
	Male	13 (54%)	6 (46%)	6 (60%)	1 (100%)
Age	Mean (±SD)	60.6 (±16.7)	60.6 (±18.4)	58.7(±14.4)	45
	Range	24 – 85	24 – 85	41 – 83	–
Race	Caucasian	13 (54%)	7 (54%)	5 (50%)	1 (100%)
	Hispanic	1 (4%)	0	1 (10%)	0
	Other	9 (37%)	6 (46%)	4 (40%)	0
**Stanford Cohort**		n = 24	n = 12	n = 7	n = 5
Sex	Female	13 (54%)	5 (42%)	5 (71%)	3 (40%)
	Male	11 (46%)	7 (58%)	2 (29%)	2 (60%)
Age	Mean (±SD)	59.0 (±16.1)	57.0 (±18.1)	54.4 (±12.1)	70.0 (±12.9)
	Range	31 - 91	32 - 91	31 - 70	57 - 86
Race	Asian	7 (29%)	3 (25%)	2 (29%)	2 (40%)
	Caucasian	11 (46%)	6 (50%)	4 (57%)	1 (20%)
	Hispanic	2 (8%)	2 (13%)	0	0
	Other	4 (17%)	1 (8%)	1 (14%)	2 (40%)

Table S2. Cohort characteristics for UOP and STA cohort, Related to Figure 1.

Adjuvant therapy	None	RTx	CRTx
**All**			
All cases (n = 24)	13	7	4
**Higher vs. lower grade**			
Lower grade (n = 12)	8	3	1
Higher grade (n = 12)	5	4	3
**WHO Grade**			
Grade 1 (n = 12)	8	3	1
Grade 2 (n = 7)	4	1	2
Grade 3 (n = 5)	1	3	1
**Recurrence within three years**			
Yes (n = 9)	4	3	2
No (n =14)	9	3	2
Not documented (n = 1)	0	1	0
**Cancer death within five years**			
Yes (n = 7)	2	3	2
No (n = 17)	11	4	2

Table S3. Adjuvant treatment and patient outcomes in the STA cohort, Related to Figure 6.
RTx = radiotherapy, CRTx = chemoradiotherapy.
